# Targeted Delivery of Mutant Tolerant Anti-Coxsackievirus Artificial MicroRNAs Using Folate Conjugated Bacteriophage Phi29 pRNA

**DOI:** 10.1371/journal.pone.0021215

**Published:** 2011-06-15

**Authors:** Xin Ye, Zhen Liu, Maged Gomaa Hemida, Decheng Yang

**Affiliations:** Department of Pathology and Laboratory Medicine, The Institute for Heart and Lung Health, St. Paul's Hospital, University of British Columbia, Vancouver, British Columbia, Canada; University of Kansas Medical Center, United States of America

## Abstract

**Background:**

Myocarditis is the major heart disease in infants and young adults. It is very commonly caused by coxsackievirus B3 (CVB3) infection; however, no specific treatment or vaccine is available at present. RNA interference (RNAi)-based anti-viral therapy has shown potential to inhibit viral replication, but this strategy faces two major challenges; viral mutational escape from drug suppression and targeted delivery of the reagents to specific cell populations.

**Methodology/Principal Findings:**

In this study, we designed artificial microRNAs (AmiRs) targeting the 3′untranslated region (3′UTR) of CVB3 genome with mismatches to the central region of their targeting sites. Antiviral evaluation showed that AmiR-1 and AmiR-2 reduced CVB3 (Kandolf and CG strains) replication approximately 100-fold in both HeLa cells and HL-1 cardiomyoctes. To achieve specific delivery, we linked AmiRs to the folate-conjugated bacterial phage packaging RNA (pRNA) and delivered the complexes into HeLa cells, a folate receptor positive cancer cells widely used as an *in vitro* model for CVB3 infection, via folate-mediated specific internalization. We found that our designed pRNA-AmiRs conjugates were tolerable to target mutations and have great potential to suppress viral mutational escape with little effect on triggering interferon induction.

**Conclusion/Significance:**

This study provides important clues for designing AmiRs targeting the 3′UTR of viral genome. It also proves the feasibility of specific deliver of AmiRs using conjugated pRNA vehicles. These small AmiRs combined with pRNA-folate conjugates could form a promising system for antiviral drug development.

## Introduction

Myocarditis, the inflammation and injury of heart muscle cells, causes up to 20% of all cases of sudden death in young adults [1]. Coxsackievirus B3 (CVB3) is the most prevalent cause of myocarditis [2] but there is still no specific cure yet. RNA interference (RNAi) has shown promising therapeutic potential against chronic viral infections [3], [4], [5]. However, it is limited by the fact that frequent mutations in the viral genome, especially the RNA virus, compromise the effect of RNAi agents (6–8). Two of the popular RNAi agents studied in anti-viral research are small interfering RNAs (siRNAs) [6], [7], [8], [9], [10], [11], [12] and artificial microRNAs (AmiRs) [5], [13], [14], the mimics of endogenous microRNAs (miRNAs). siRNAs function by binding perfectly to the recognition sites within viral genomic RNA or mRNA and cleaving those molecules [15], while AmiRs usually partially bind to their targets and destabilizing them or inhibit their translation. However, one of the greatest challenges to develop specific and effective RNAi-based antiviral therapy is that some viruses, especially RNA virus like CVB3, possess a very high mutation rate [16], [17], [18], [19]. siRNA treatment itself can also trigger vial mutations sometimes [20], [21], [22]. Merl and others found that CVB3 can generate mutants resistant to siRNA targeting [23]. Although targeting the conserved region of viral genome [24] or treating the cells with a combination of several siRNAs [23], [25] can minimize the viral escape, there are still viral mutants appeared and the viral titer restored to relatively high level at 140 h post infection (pi) [23]. Different from siRNAs, AmiRs are expressed in the form of primary miRNAs in the nucleus and cleaved by Drosha and DGCR8 into the precursor miRNAs (pre-miRNAs), which are transported into cytoplasm and further processed by Dicer into ∼21-nt miRNA duplexes with two imperfect complementary strands [26], [27], [28]. One, sometimes both of the two strands would act as the mature miRNA incorporated into the RNA-induced silencing complex (RISC). AmiRs target to, under most circumstances, the 3′untranslated region (UTR) of selected mRNAs with partial complementary sequences, resulting in the degradation or translational inhibition of the targets [29]. Some of the AmiRs can form fewer than 10 base-pairs (only half of the total length of miRNAs) with the targeting sites, making them better able to tolerate mutations [30]. Though siRNA treatments against CVB3 infection have been tested in several studies [10], [23], [24], [25], [31], [32], [33], there is still no report on using AmiRs to inhibit CVB3 infection and replication. Considering that AmiRs have better mutation tolerance to their targets and are less toxic than traditional RNAi methods [34], [35], it is worthwhile to design AmiRs against CVB3.

**Figure 1:**
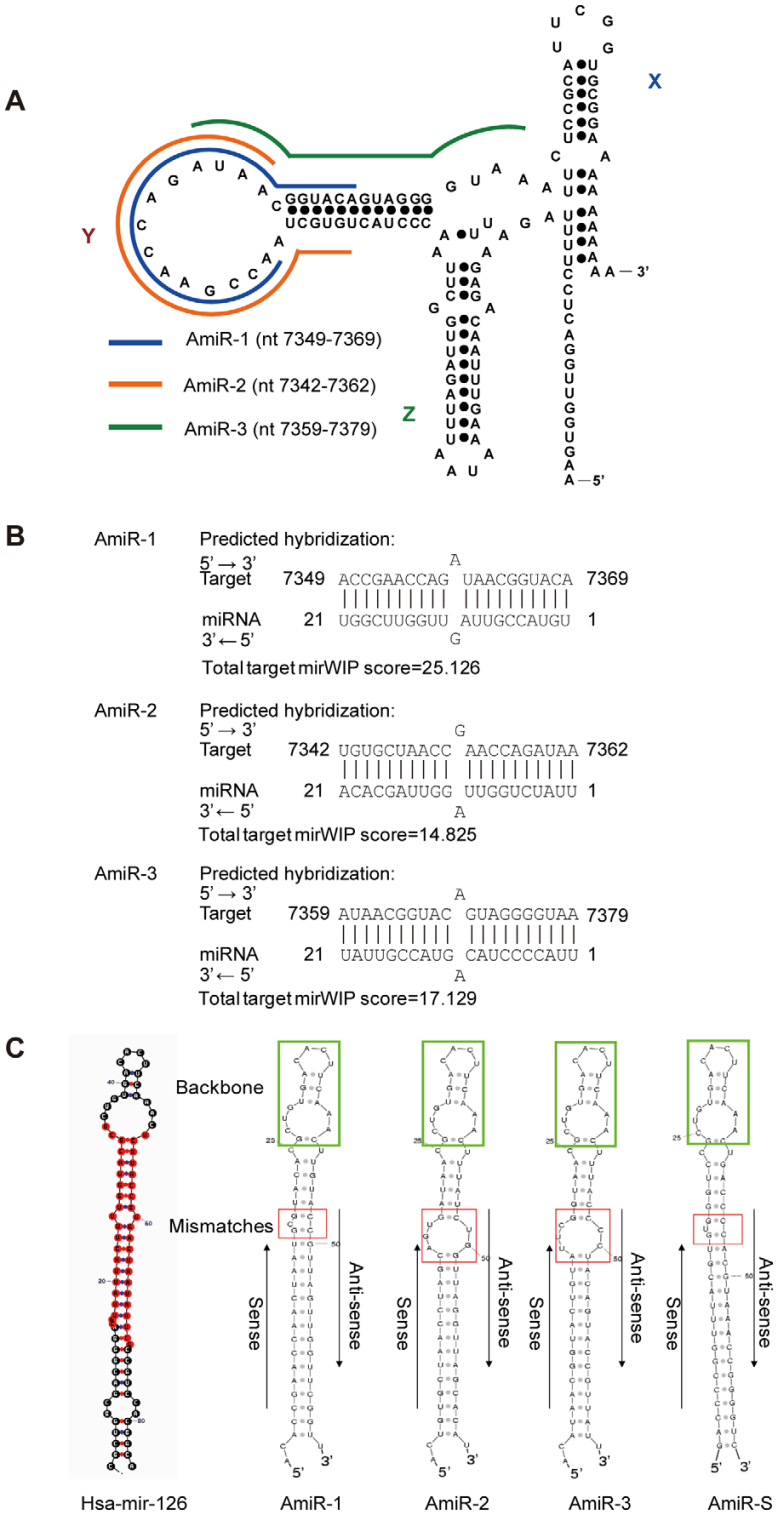
Design of anti-CVB3 AmiRs A) AmiR targeting sites in the 3′UTR of CVB3 genome. The secondary structure of 3′UTR of CVB3 contains three stem-loops, X, Y and Z. Three AmiR candidates, AmiR-1 (blue), AmiR-2 (orange) and AmiR-3 (green), were designed to target the stem-loop Y, the biggest stem-loop. **B) The predicted complementation between AmiRs and their target sequences.** The prediction was conducted by using Sfold software. The total target mirWIP scores are indicated. A mismatch was designed in the middle of the target. **C) Secondary structures of pre-AmiRs.** All the AmiRs were inserted to the hsa-mir-126 scaffold to form pre-miRNAs. The sequences circled by the green box are backbones of miR-126; sense and antisense strand of AmiR sequences were labeled. The mismatch regions between the two strands were marked with red box.

The CVB3 genome encodes a single long open reading frame flanked by a 5′ and 3′UTR [36]. The secondary structure of CVB3 3′UTR contains three stem-loops (Fig. 1A) [37]. The interactions among these stem-loops enable the formation of kissing-pair tertiary structure facilitating viral translation and replication [37]. Some host cell proteins, such as La autoantigen in HeLa cells, can bind to the 3′UTR of CVB3, affecting viral replication [38]. Our group designed antisense oligodeoxynucleotides (AS-ODNs) targeting the 3′UTR and inhibited the translation and replication of CVB3 [39]. All of the evidence supports the vital roles of the 3′UTR in the replication of CVB3. In addition, most endogenous miRNAs regulate gene expression by targeting the 3′UTR [29]. There have been studies using AmiRs targeting the 3′UTR of animal genes [40], [41]. Liu and coworkers designed AmiRs targeting the coding region of HIV and they found these AmiRs also targeted the 3′UTR of HIV genome which was proved by luciferase assay [5]. However, designing AmiRs targeting 3′UTR viral genome is still a relatively new strategy. Therefore, there is some prospect of developing AmiRs to target the 3′UTR of CVB3.

Another barrier to RNAi-based therapy is the non-specific distribution of the drug *in vivo* after administration. Our group and others have used a novel approach to deliver siRNA to inhibit viral replication [42] or oncogenesis [43], [44], [45] with ligand-linked bacterial phage packaging RNA (pRNA) as a vehicle, but this method has not been tested on AmiRs. Discovered about two decades ago within the bacteria phage 29, pRNA plays essential roles in packaging the phage DNA [46] and has great potential to deliver nucleic acid based drugs. pRNA monomers are also capable of assembling dimmers, trimers and even hexamers [47], [48], [49], [50], which makes it possible to deliver drug combinations conveniently. This multimerization may also enable the pRNA to carry both therapeutic molecules and the ligand simultaneously for targeted delivery. Previous studies have demonstrated that the folate-conjugated pRNA is a powerful vehicle to deliver siRNA molecules into the cancer cell lines [42], [43], [44], [45]. Folate is an essential extrinsic vitamin required by most mammalian cells for nucleotide biosynthesis and thereby important for DNA synthesis and repair. The folate receptor is one of the receptors responsible for transport of folate and is highly expressed in epithelial cells, macrophages and various tumor cells like HeLa cells [51], [52], [53]. Thus folate is a promising ligand candidate for the targeted drug delivery to cancer cells.

The main goal of this study was to design effective AmiRs with mutation tolerance to inhibit CVB3 replication. Further, we investigated the feasibility to deliver AmiRs using folate-linked pRNA as a vehicle. These AmiRs linked with the pRNA were delivered into the cells either by Oligofectamin™ mediated transfection or by folate mediated internalization and the antiviral effect was evaluated. We report that AmiRs targeting the stem-loop of the CVB3 3′UTR are tolerable to target mutations and effective in inhibiting replication of CVB3 with little effect on interferon (IFN) induction. Therefore, the pRNA-ligand mediated delivery strategy holds promise for more specific delivery of AmiRs to treat viral infection.

## Materials and Methods

### Virus, cell culture, infection and transfection

CVB3 (Kandolf) was produced from a full-length cDNA clone (provided by Reinhard Kandolf, University of Tubingen, Germany) and CVB3 (CG strain) was provided by Charles J. Gauntt. Viruses were stored at −80°C. HeLa cells (American Type Culture Collection), the viral hosts, were cultured in a Dulbecco's modified Eagle's medium (DMEM) (Lonza) supplemented with 10% fetal bovine serum (FBS) (Sigma) and 100 µg/ml of penicillin-streptomycin (Invitrogen) in a 37°C incubator containing 5% CO_2_. HL-1 cells, a cardiomyocyte cell line as another cell host, were obtained from Dr. William C. Claycomb. (Louisiana State University Medical Center, New Orleans, LA) and maintained as described previously [54]. Virus was amplified in HeLa cells by infection. Virus titers were routinely determined at the beginning of each experiment by plaque assay as described later. For infection, 8×10^5^ cells were seeded to each well of a 6-well plate. After incubation at 37°C for 20 h, cells were washed with phosphate buffered saline (PBS) (Lonza) and infected with CVB3 (Kandolf or CG) at a multiplicity of infection (MOI) of 0.01 or 10 for 60 min. Sham infection was conducted using supernatant without CVB3 as a negative control. After infection, the cells were washed with PBS, overlaid with 2 mL of DMEM containing 10% FBS, and incubated for 24 h or 8 h at 37°C. Finally, the supernatants from each treatment were collected by centrifugation at 5,000× g for 5 min and kept at −80°C until use. The cell pellets were re-suspended in a Modified Oncogen Science lysis buffer (MOSLB) (50 mM NaPyrophosphate, 50 mM NaF, 50 mM NaCl, 5 mM EDTA, 5 mM EGTA, 100 µM Na3VO4, 10 mM HEPES, and 0.1%Triton X-100) and the cell lysates were stored at −20°C until use.

The transfection of pRNA-AmiRs was conducted by using Oligofectamine™ (Invitrogen) following the manufacturer's instructions with minor modifications. Briefly, HeLa cells or HL-1 cells (3×10^5^) were seeded to each well of a 6-well plate for 24 h. When cells reached approximately 50% confluence, they were washed with PBS and incubated with 1 mL of transfection complexes containing 3 µL of Oligofectamine™ and 100 pmol pRNA-AmiRs for 48 h. Mock transfection was conducted using the Oligofectamine™ without pRNA-AmiRs. Cells were then infected with CVB3 (Kandolf or CG) as described above.

### AmiR design

Three 21-nt AmiRs (AmiR-1, AmiR-2, AmiR-3) with one nucleotide mismatches to their targets in the middle region (nt 11^th^) were designed to target the 3′UTR of CVB3 (Fig. 1A, 1B) and one scrambled AmiR (AmiR-S) was used as a negative control. For each AmiR, we also designed a partial complementary strand to form double-stranded AmiR duplexes containing wobble base-pairing mimicking the endogenous miRNA duplexes (Fig. 1C). All AmiRs were incorporated to a human hsa-miR-126 precursor backbone to form pre-AmiRs mimicking the *in vivo* pre-miRNAs (Fig. 1C). Each pre-AmiR was synthesized along with the 5′ and 3′ end sequences containing restriction enzyme sites BamHI and AflII, respectively. These segments were inserted into a miRNA-expressing vector, pRNAT-CMV 3.1/Hygro (Genscript), which carries a cGFP (coral GFP) reporter gene. The AmiR sequences were confirmed by sequencing.

### Establishment of AmiR-expressing HeLa cell lines

AmiR-expressing HeLa cell lines, which can grow well in the presence of 150 µg/mL hygromycin, were established by plasmid transfection using the Lipofectamine™ 2000 reagent following the manufacturer's instructions (Invitrogen). Briefly, HeLa cells (∼1.5×10^6^) were seeded in each 60-mm culture dish. After incubation for 20 h, the cells reached about 90% confluence and were washed twice with PBS. A transfection mixture containing 8 µg of plasmid DNA and 20 µL of Lipofectamine™ 2000 in 1 mL of Opti-MEM® (Invitrogen) was added to each dish and the cells were incubated for 6 h at 37°C. The cells were then washed with PBS and incubated with 4 ml of DMEM containing 10% FBS at 37°C. After transfection for 24 h, cells were washed with PBS, lifted from plates by Trypsin-EDTA (Gibco) and subcultured to several 10-cm plates with 10 mL of DMEM containing 10% FBS and 150 µg/mL hygromycin for each plate. The medium was changed every 48 h. After about 2 weeks, the survived clones appeared were passaged to a 24-well plate and checked under fluorescent microscope (Nikon). The green ones were further confirmed by Western blot detection for the expression of cGFP.

### Preparation of pRNA-AmiRs

The pRNA-AmiR chimerics were produced by *in vitro* transcription of cDNA fragments encoding the corresponding pRNA-AmiRs. The cDNA fragments were amplified by PCR using the primers listed in Table 1 and a pRNA A-b' or pRNA B-a' plasmid kindly provided by Dr. Peixuan Guo [55] as templates. The *in vitro* transcription was driven by the T7 phage 2.5 promoter using T7-MEGAShortscript kit (Ambion) according to the manufacturer's instructions. 300 ng of the amplified cDNA fragments were transcribed in a 40 µL reaction volume in the presence of ATP, CTP, GTP and UTP (7.5 mM each) at 37°C for 4 h. The transcription products were then purified by 8% urea-polyacrylamide gel electrophoresis (PAGE) in TBE buffer and eluted with 0.5 M sodium acetate, 0.1 mM EDTA and 0.1% sodium dodecyl sulfate (SDS). The eluted products were further precipitated with ethanol and re-suspended in RNase free water.

**Table 1:** Primers used for producing pRNA-AmiR chimerics and luciferase reporter constructs.

DNA	Primer Sequences (5′→3′)*
pRNA vector	Forward: TAATACGACTCACTATTAGGGTACGGTACTTCCATTGTCATGTGTATGTTGGGGATTA
	Reverse: TGCACTTTTGCCATGATTGACGGACAATCAAC
pRNA-miRNA1	Forward: TAATACGACTCACTATTAACCGAACCAACTAATGCGTACATTTGTATGTTGGGGATTA
	Reverse: ACCGAACCAACTAACGGTACAAAATTGACACGCAATCAAC
pRNA-miRNA2	Forward: TAATACGACTCACTATTATGTGCTAACCTAGCAGTGATAATTTGTATGTTGGGGATTA
	Reverse: TGTGCTAACCTAACCAGATAAAAATTGACACGCAATCAAC
pRNA-miRNAS	Forward: TAATACGACTCACTATTAGACCCCGGTTTACGTGTGGGTCTTTGTATGTTGGGGATTA
	Reverse: GACCCCGGTTTACGTGGGGTCAAATTGACACGCAATCAAC
Wt-CVB3-3UTR	Forward: GCCG*GTTTAAAC*GACAATTTG
	Reverse: GCGCG*TCTAGA*TTTTTTTTTTCC

*The T7 promoter regions are underlined and the restriction enzyme sites are italicized.

### Labeling of pRNA-AmiR chimerics with folate

Folate-labeled pRNA-AmiR chimerics were prepared as previously described for labeling pRNA-siRNA [42], [45]. 650 ng (0.2 µM in final concentration) of cDNA fragments were transcribed in 40 µl of reaction buffer containing 40 mM Tris (pH 8.0), 6 mM MgCl_2_, 2 mM spermidine, 0.01% Triton X-100, 5 mM DTT, and 5 U/µL T7 RNA polymerase (Promega), together with 4 mM folate-AMP (synthesized by TriLink BioTechnologies, USA), 0.25 mM ATP, 1 mM CTP, GTP and UTP. The transcription products were then purified by 8% urea-PAGE, eluted with 0.5 M sodium acetate, 0.1 mM EDTA and 0.1% SDS and analyzed by separation through an 8% urea-PAGE in TBE buffer. About 1 µg RNA in gel loading buffer II (Ambion) was heated at 95°C for 5 min and then loaded onto the gels. Gels were run at 140 V for 2–2.5 h and then stained in TBE buffer containing 3× Gel Read (Biotium).

### Folate-pRNA-AmiR heterodimer formation

Folate-labeled pRNA-AmiR heterodimers were prepared by mixing folate-pRNA-Ba' and pRNA-Ab' (AmiR-1), or pRNA-Ab' (AmiR-2), or pRNA-Ab' (AmiR-S) which are based on a pRNA (A-b') structure in a 1∶1 molar ratio (0.2 µM each) in a total volume of 200 µL solution containing 10 mM MgCl_2_, 5 µL of RNAse OUT RNase inhibitor (Invitrogen), 15 µL of 0.1 mM DTT and 20 µL of 10× RT buffer (Invitrogen) and then incubated at 37°C for 1 h. The pRNA heterodimers were analyzed by native PAGE in TBM buffer as described previously [56].

### Delivery of folate labeled pRNA-AmiR monomers or heterodimers into HeLa cells

HeLa cells were maintained in folate-free RPMI 1640 medium (Gibco) for two weeks and 8×10^5^ cells/well were then plated into a 6-well plate one day before the experiment. Cells were then washed with PBS containing 10 mM MgCl_2_ and incubated with 0.5 nmol of folate-labeled pRNA-AmiR monomers or heterodimers in 1 mL of binding solution (serum free RPMI medium containing 10 mM MgCl_2_, and 1 U/µL SUPERRNase inhibitor (Ambion)) for 8 h at 37°C. After incubation, free RNA was removed by washing with PBS. Mock treatment using the same binding solution only was conducted to serve as a negative control. Cells were then infected with CVB3 (Kandolf) at 0.01 MOI for 20 h. Cell lysates and supernatants were collected for Western blot and viral plaque assay to evaluate the CVB3 replication as described below.

### Quantitative reverse-transcriptional PCR (q-RT-PCR)

Total RNAs from AmiR-expressing cell lines or pRNA-AmiR transfected cells were isolated using miRNeasy Mini Kit (Qiagen). The RNAs were then analyzed by q-RT-PCR using Custom TaqMan Small RNA Assay reagents (Applied Biosystems, Assay ID : pRNA-AmiR-1: CSY9XW6; pRNA-AmiR-2: CS0IV3E) based on the stem-loop RT-PCR technology, which only measures the mature form of AmiRs [57]. An U6 snRNA measurement using TaqMan Assay served as an endogenous control.

### Cell viability assay

Cell viability was measured by using a 3-(4,5-dimethylthiazol-2-yl) -5-(3-carboxymethoxyphenyl)-2-(4-sulfophenyl)-2H-tetrazolium salt (MTS) assay kit (Promega) according to the manufacturer's instructions. Cells were incubated with MTS solution for 2 h, and the absorbance was measured at 492 nm using an enzyme-linked immunosorbent assay (ELISA) reader (SLT Lab Instruments). The survival values for absorbance of sham-infected cells were defined as 100%, and the remaining infected data, including that for AmiR-expressing cells, pRNA-AmiR transfected cells, folate-pRNA-AmiR monomer or dimmer treated cells and mock-transfected cells, was converted to the ratio of the sham-infected sample. Morphological changes of cells following CVB3 infection were evaluated by phase-contrast microscopy.

### Western blotting analysis

Protein concentration for each sample was quantified by the Bradford assay using fat-free-bovine serum albumin (BSA) as the standard. Western blotting was performed by standard protocols as previously described [39]. Briefly, equal amounts of proteins were subjected to sodium dodecyl sulfate (SDS)-PAGE and then transferred to nitrocellulose membranes (Pall Corporation). The membranes were blocked with 5% skim milk in TBST buffer (25 mM Tris-HCl, 137 mM NaCl, 0.1% (v/v) Tween-20, pH 7.6) for 1 h and probed with primary monoclonal mouse antibodies against CVB3 capsid protein VP-1 (DAKO), beta actin (Sigma), or caspase-3 (Santa Cruz) or a primary rabbit polyclonal antibody against GFP (Invitrogen) overnight at 4°C, followed by wash with TBST for three times and incubation with horseradish-peroxidase-conjugated goat secondary antibody to anti-mouse immunoglobulin G or to anti-rabbit immunoglobulin G (1∶2000 dilution, Santa Cruz) for 90 min at room temperature. The signals were detected by ECL reagents (Syngene).

### Viral plaque assay

The virus titer was determined by plaque assay as described previously [58]. Briefly, HeLa cells (8×10^5^ cells/well) were seeded into 6-well plates and incubated at 37°C for 20 h. When cell confluence reached approximately 90%, cells were washed with PBS and then overlaid with 800 µl of diluted supernatants containing viral particles. The cells were incubated at 37°C for 60 min, and the supernatants were removed. Finally, cells were washed with PBS again and overlaid with 2 ml of sterilized soft Bacto-agar-minimal essential medium (0.75% Agar). The cells were incubated at 37°C for 72 h, fixed with Carnoy's fixative for 30 min, and then stained with 1% crystal violet. The plaques were counted, and the virus titer was calculated as the plaque forming units per ml (pfu/mL).

### Luciferase reporter assay

Wild type (wt) CVB3 3′UTR (nt 7306–7399 GenBank: M88483.1) was cloned from a previous CVB3 3′UTR clone [38] by PCR using primers including the restriction enzyme sites listed in Table 1. The mutant (mut) CVB3 3′UTRs (Mut-Y1 and Mut-Y2) containing the restriction enzyme sites were synthesized using miniGENES synthesis service from Integrated DNA Technologies. Each 3′UTR fragments (wt or mut) was inserted at the PmeI and XbaI restriction sties into the pmirGLO Dual-Luciferase miRNA Target Expression Vector (Promega) which co-expresses firefly and *Renilla* luciferases. For luciferase reporter assay, HeLa cells (2×10^4^/well) were plated in 96-well plates for 24 h and then co-transfected with the constructed luciferase reporter plasmids (wt or mut, 100 ng/well) and the pRNA-AmiR chimerics (2 pmol/well) using Lipofectamine™ 2000 (Invitrogen). Cells co-transfected with pRNA-AmiR chimerics and the pmirGLO empty vector or only transfected with a reporter plasmid vector were used as controls. Two days post transfection, firefly and *Renilla* luciferase activities were detected on an ELISA reader (TECAN) using the Dual-Glo luciferase analysis system (Promega) according to the manufacturer's protocol. The relative luciferase activity was calculated using the ratio of firefly to *Renilla* and then normalized to the pmir-GLO control group which was set as 1.0.

### Interferon (IFN) induction analysis

Total RNAs from HeLa cells transfected with AmiR-expression vectors or pRNA-AmiR were collected using miRNeasy Mini Kit (Qiagen). The RNAs were reverse transcribed to cDNA using SuperScript® III First-Strand Synthesis System (Invitrogen). The gene markers for interferon induction including IFITM1, ISGF3-gamma, OAS1 and OAS2 were detected using the Interferon Response Detection Kit (System Biosciences) by q-PCR and normalized to beta-actin following the manufacturer's instructions. The Oligofectamine™ transfected HeLa cells were used to serve as a negative control, and the positive control (cDNA from IFN treated cells) was provided by the kit.

### Statistical analysis

Statistical significance was evaluated using the Student *t*-test for paired comparisons. All values are expressed as means ± SD. A *p*-value of <0.05 was considered statistically significant.

## Results

### Design of AmiR sequences targeting the 3′UTR of CVB3

The 3′UTR is an essential region of CVB3 genome for its replication [37], [38] and most endogenous miRNAs exert their inhibitory effect by binding the 3′UTR of the target mRNAs [59]. These reports led us to design three AmiRs targeting the CVB3 3′UTR (Fig. 1A). Among the three (X, Y and Z) loop structures of CVB3 3′UTR, Y loop was selected for AmiR targeting (AmiR-1 targeting nt 7349–7369 and AmiR-2 targeting nt 7342–7362) for the following two reasons; First, the interaction between loop X and Y was shown to be critical for CVB3 replication [37]; Secondly, being the largest loop structure, Y loop may provide more space to allow the AmiRs to bind to the single strand region more easily. An on-line prediction tool STarMir (http://sfold.wadsworth.org/cgi-bin/starmir.pl) was used to screen potential AmiRs targeting the different regions of CVB3 3′UTR and those targeting the Y loop region scored the highest (Fig. 1B). We also designed one AmiR targeting the stem region close to the Y loop (AmiR-3 targeting nt 7359–7379) for comparisons and one scrambled AmiR (AmiR-S) as a negative control.

In order to mimic the endogenous miRNA strucrure, we introduced mismatches between the designed AmiRs and their potential targets. All the mismatches were located in the middle region of the AmiRs to follow the general miRNA targeting principles published previously [30], [60], [61]: i) the 5′ end regions of miRNAs (nt 2-7 or -8), named as the seeding region, are mandatory to be complementary to the targets, ii) the presence of highly complementary sequences in the 3′ end region of miRNAs improves the specificity of miRNAs and iii) the miRNAs with mismatches to their targets in the central region (nt 9–11) are more likely to be effective than the ones with mismatches in other regions. A partial complementary strand was also designed for each AmiR to form a double-stranded molecule (miRNA duplex) similar to natural miRNAs (Fig. 1C). In order to mimic the *in vivo* process of miRNA, the AmiRs were incorporated into a natural miRNA precursor backbone. Previous studies have shown that both animal and plant miRNA precursors can be modified to express small RNAs with sequences unrelated to the original miRNA produced by the precursors [62], [63]. In our study, we used human hsa-mir-126 precursor (Fig. 1C), which has been proved to be effective for AmiR construction [64], for AmiR expression in HeLa cell lines.

### Inhibition of CVB3 replication by AmiRs

**Figure 2:**
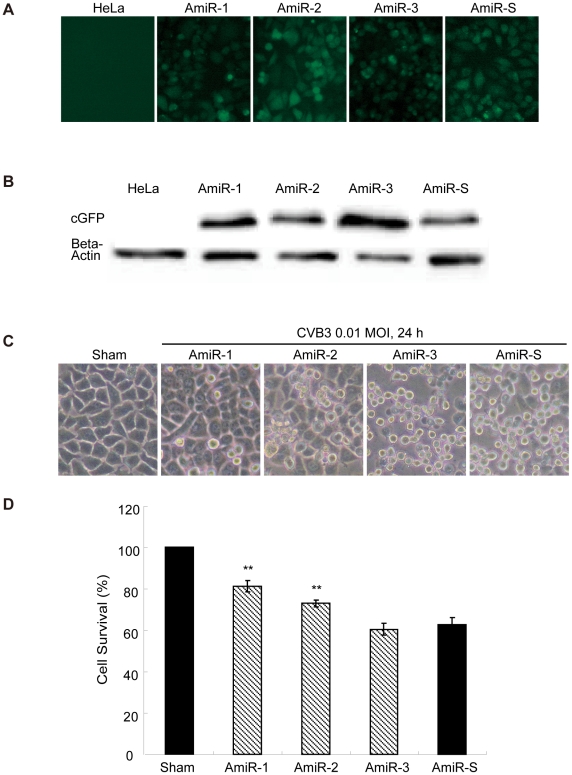
AmiRs inhibit CVB3 induced cell death in AmiR-expressing cell lines. **A) Establishment of AmiR-expressing cell lines.** Four stable cell lines over-expressing an individual AmiR were established by transfection of HeLa cells with the AmiR plasmids. cGFP tag expression was used as a selection marker. **B) Western Blotting analysis for cGFP expression of the established AmiR cell lines.** The cell lysates of these cell lines were collected and applied to Western blotting detection of cGFP expression. The non-transfected HeLa cell lysate served as a negative control. **C) Cell morphology changes after CVB3 infection.** Cell lines expressing different AmiRs were infected with CVB3 (Kandolf). Cell morphology was observed under a phase-contrast microscope. Dying cells appeared rounding and detachment. **D) MTS assay of CVB3 infected AmiR cells.** Cell lines expressing different AmiRs were infected with CVB3 as described above. Cell viability was measured by MTS assay. The survival values of the samples were converted to the ratio of the sham-infected HeLa cells, which were defined as 100%.

**Figure 3:**
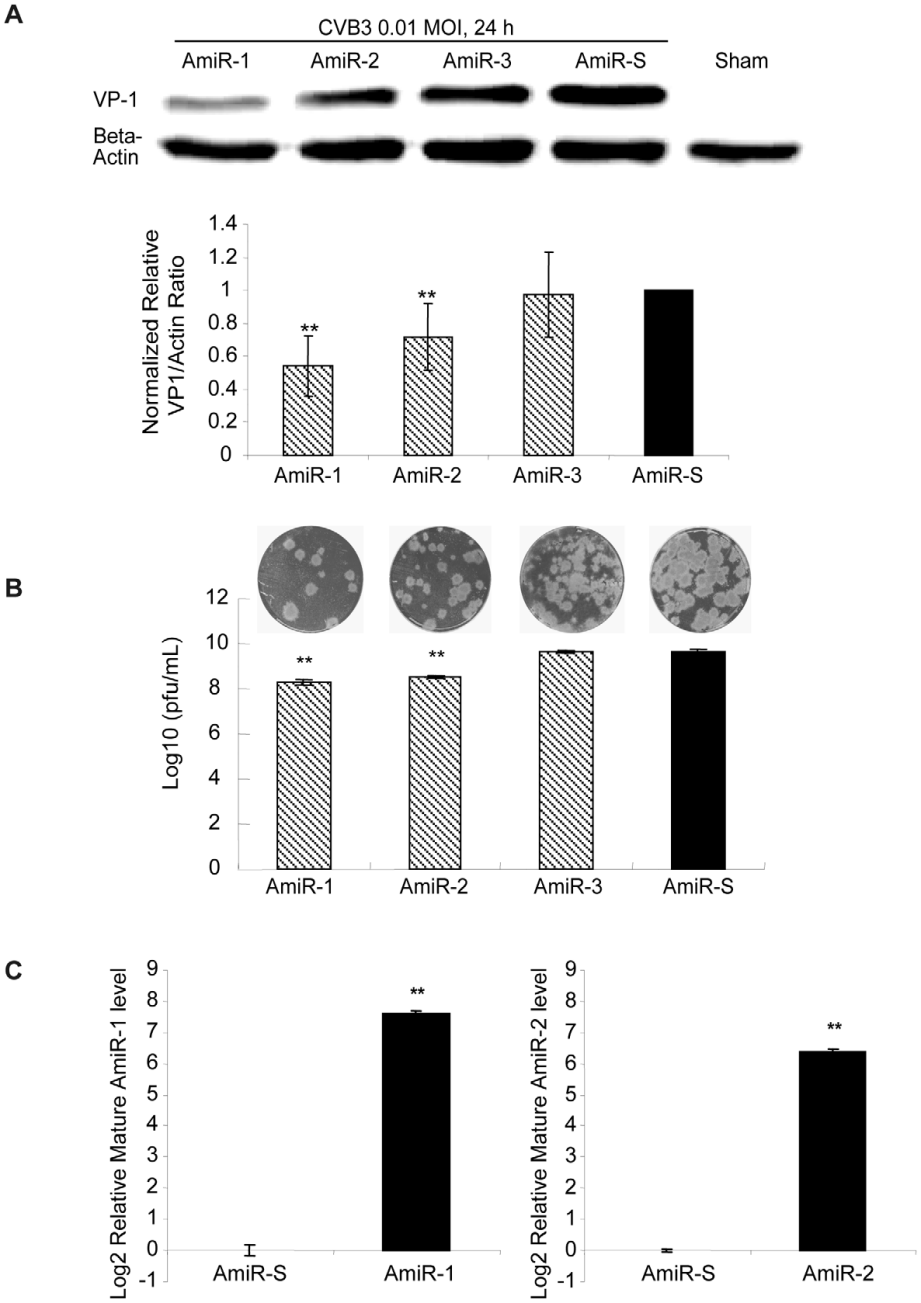
AmiRs inhibit CVB3 replication in AmiR-expressing cell lines. **A) Western blotting detection of CVB3 VP-1 protein.** Different AmiR cell lines were infected with CVB3 (Kandolf) and cell lysates were collected for VP-1 detection by Western blotting. Sham infected cells served as a negative control. The VP-1 expression levels were quantified by densitometry and normalized to the level of beta-actin. The ratios of VP-1 to beta-actin were further converted to the ratios of the value of AmiR-S cells, which was set as 1.0. **B) Viral plaque assay.** Cells were infected with CVB3 at 0.01 MOI for 24 h. The supernatant was collected to perform viral plaque assay to determine the viral titer. The upper panel is the representative viral plaques for each cell line and the lower panel is the calculation of viral titers. **C) q-RT-PCR detection of mature AmiRs.** Total RNAs from the AmiR-1, AmiR-2 and AmiR-S cell lines were extracted. The expression of mature AmiR-1 and AmiR-2 were measured by q-RT-PCR and normalized to the expression of U6 RNA in each sample. The relative mature AmiR-1 and AmiR-2 levels in the AmiR-S cell line were set as 0. The values shown represent the means ± SD of the data from three independent experiments, *P*<0.05.

To test if our designed AmiRs could inhibit CVB3 replication, HeLa cell lines expressing these individual AmiRs were established by transfection of the cells with the constructed AmiR expressing plasmids. Positive cell colonies were confirmed by fluorescent microscopy and Western blot detection of cGFP expression (Fig. 2A and 2B). The different cell lines were subsequently infected with CVB3 (Kandolf) and the antiviral effect of the AmiRs was evaluated by observation of cell morphologies and measurement of cell viabilities. As shown in Fig. 2C, more than 80% of AmiR-1 expressing cells were still alive after 24 h infection. The anti-CVB3 activity was followed by AmiR-2 (more than 70% cell survival). However, AmiR-3 did not show significant protection of the cell against CVB3 infection compared with AmiR-S control. The result of MTS assay was consistent with the morphological observation (Fig. 2D). To investigate the impact of AmiRs on CVB3 gene translation, we determined the expression levels of CVB3 VP-1 by Western blot. Fig. 3A shows that the VP-1 levels were significantly lower in the AmiR-1 (0.55) and AmiR-2 (0.70) cells than in the control (1.00). However, AmiR-3 did not show significant inhibition of CVB3 VP1 synthesis. The antiviral effect of these AmiRs was further validated by viral plaque assay. Our results confirmed that AmiR-1 and AmiR-2 reduced the viral particle numbers by approximately 1.5 log_10_ and 1.2 log_10_, respectively; whereas AmiR-3 did not lower the viral replication as compared with the AmiR-S control group (Fig. 3B).

To confirm that the antiviral effects of AmiR-1 and AmiR-2 were due to the expression of mature AmiRs in the established cell lines, we conducted q-RT-PCR to measure the expression levels of mature AmiR-1 and AmiR-2 and demonstrated an over 300-fold increase of these mature AmiRs in the corresponding cell lines as compared to the AmiR-S control (Fig. 3C). As expected, the AmiR-1 or AmiR-2 level was undetectable in the AmiR-S cell line. This demonstrated that the designed AmiRs were properly processed into mature and functional forms in the cells.

### pRNA-AmiRs chimerics can be processed into mature functional AmiRs

**Figure 4:**
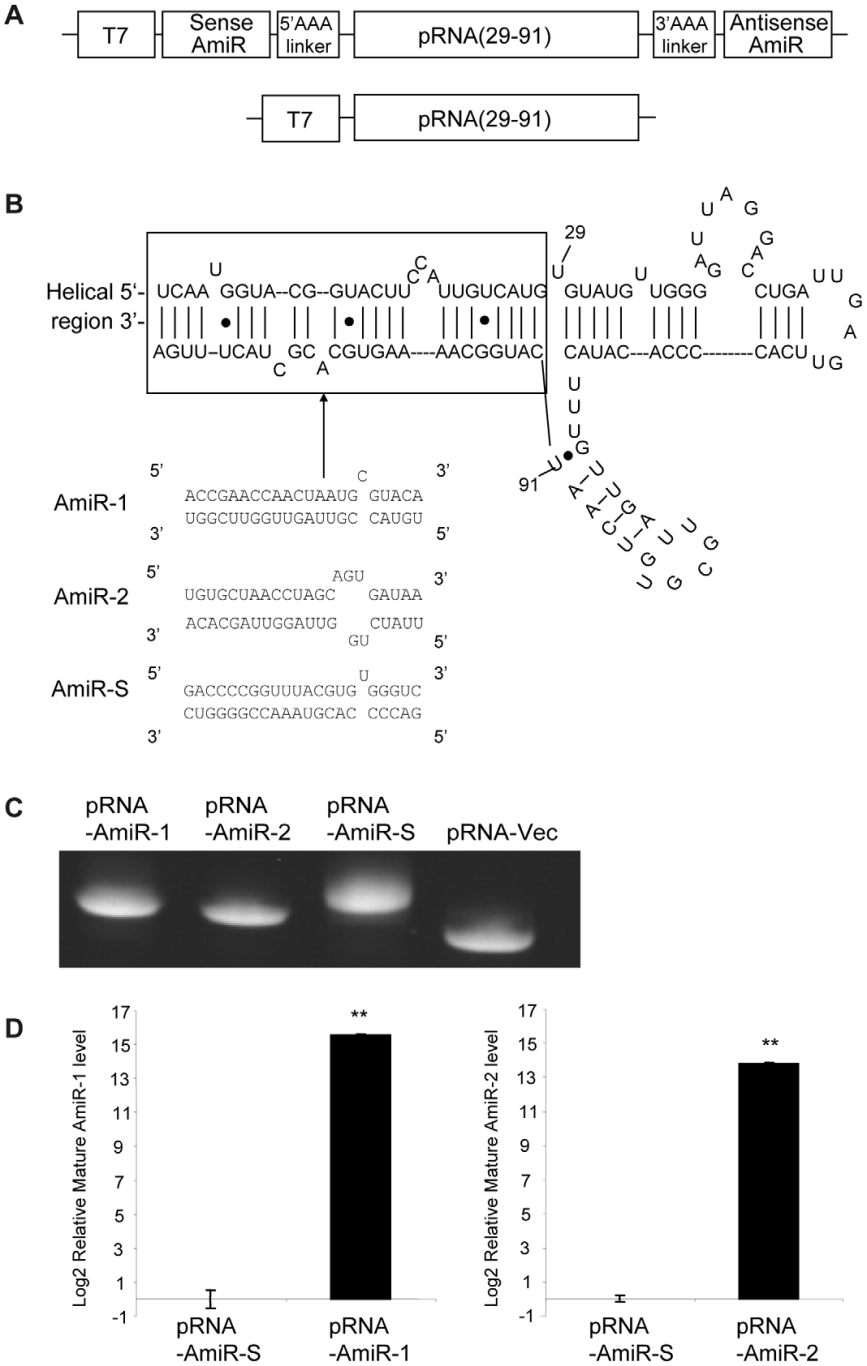
Preparation of pRNA-AmiR chimerics. **A) Schematic structure of cDNA encoding pRNA-AmiR chimerics or pRNA-Vector.** Sense, antisense AmiRs and the pRNA backbone were connected by poly-A linkers. The transcription of pRNA-AmiR chimerics was under the control of a T7 phage 2.5 promoter. **B) Phage 29 pRNA sequence and its secondary structure.** The double stranded helical region marked by the box would be replaced by the AmiRs sequences listed below to form pRNA-AmiR chimerics. **C) Analysis of pRNA-AmiR chimeric transcripts.** The three chimeric pRNA-AmiRs and pRNA-Vec were analyzed by denaturing urea-PAGE. **D) q-RT-PCR detection of mature AmiR.** HeLa cells were transfected with pRNA-AmiRs for 48 h and the expression levels of mature AmiR-1 and AmiR-2 were measured by q-RT-PCR using total RNAs and normalized as described in Figure 3. The values shown represent the means ± SD of the data from three independent experiments, *P*<0.05.

In order to improve the targeted delivery of the AmiRs, the designed AmiRs were linked to the pRNA vehicle by *in vitro* transcription of pRNA plasmid DNA (Fig. 4A) [55] using primers listed in Table 1. The produced RNA transcripts containing, in order, the sense AmiR sequence, a 5′ poly-A linker, the pRNA vector sequence (nt29–91), a 3′poly-A linker and the anti-sense AmiR sequence. The secondary structure of the pRNA-AmiR is shown in Fig. 4B. The three AmiR sequences listed in the lower panel were used to replace the helical region of the pRNA as indicated in the box. These chimeric transcripts were purified with urea-polyacrylamide gel running in TBE buffer (Fig. 4C). A pRNA-Vec (nt 29–91 of original pRNA sequence) was prepared as a negative control.

To ensure that the AmiRs had been properly processed into mature form after delivery to the cells, we performed q-RT-PCR to detect the mature AmiR levels in the pRNA-AmiR transfected HeLa cells. Fig. 4D shows that the mature AmiR-1 and AmiR-2 levels were dramatically increased in the pRNA-AmiR-1 and pRNA-AmiR-2 transfected cells, respectively, compared with the pRNA-AmiR-S treated cells. This indicates that the AmiRs were properly processed in the cells.

**Figure 5:**
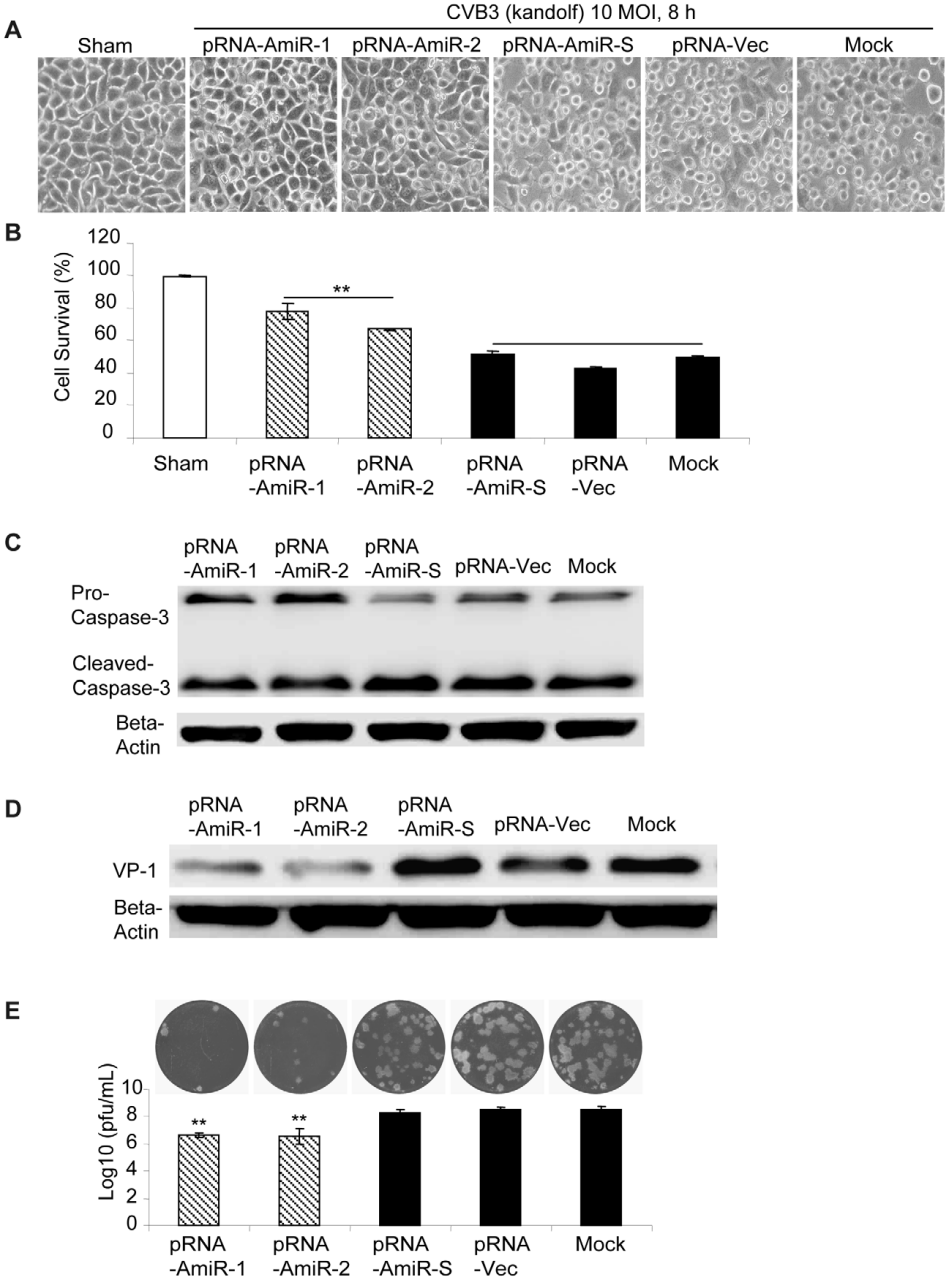
Anti-CVB3 (Kandolf, high MOI) effect of pRNA-AmiR chimerics in HeLa cells. HeLa cells were transfected with different pRNA-AmiRs or mock-transfected with Oligofectamine™ only for 48 h and then infected with CVB3 (Kandolf, 10 MOI 8 h) or sham-infected with PBS. Cell morphologies were analyzed by phase-contrast microscopy. Dying cells appeared rounding and detachment (A). Cell viability was measured by MTS assay (B), the viral VP-1 protein and caspase-3 cleavage was detected by Western blot analysis (C, D) and viral particle formation was measured by plaque assay (E).

**Figure 6:**
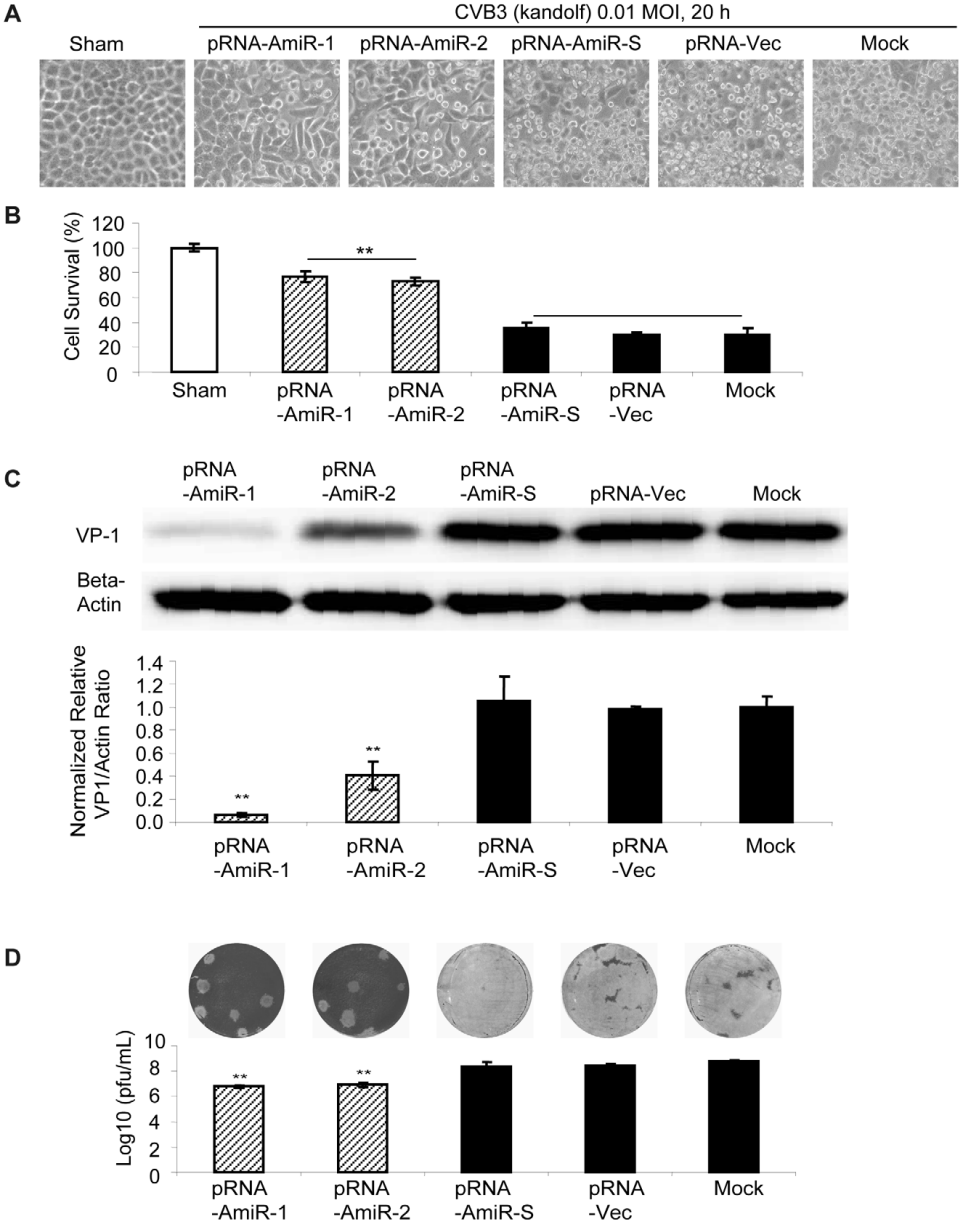
Anti-CVB3 (Kandolf, low MOI) effect of pRNA-AmiR chimerics in HeLa cells. HeLa cells were transfected with different pRNA-AmiRs or mock-transfected with Oligofectamine™ only for 48 h and then infected with CVB3 (Kandolf, 0.01 MOI 20 h) or sham-infected with PBS. Cell morphologies were analyzed by phase-contrast microscopy. Dying cells appeared rounding and detachment (A). Cell viability was measured by MTS assay (B), the viral VP-1 protein was detected by Western blot analysis (C) and viral particle formation was measured by plaque assay (D).

**Figure 7:**
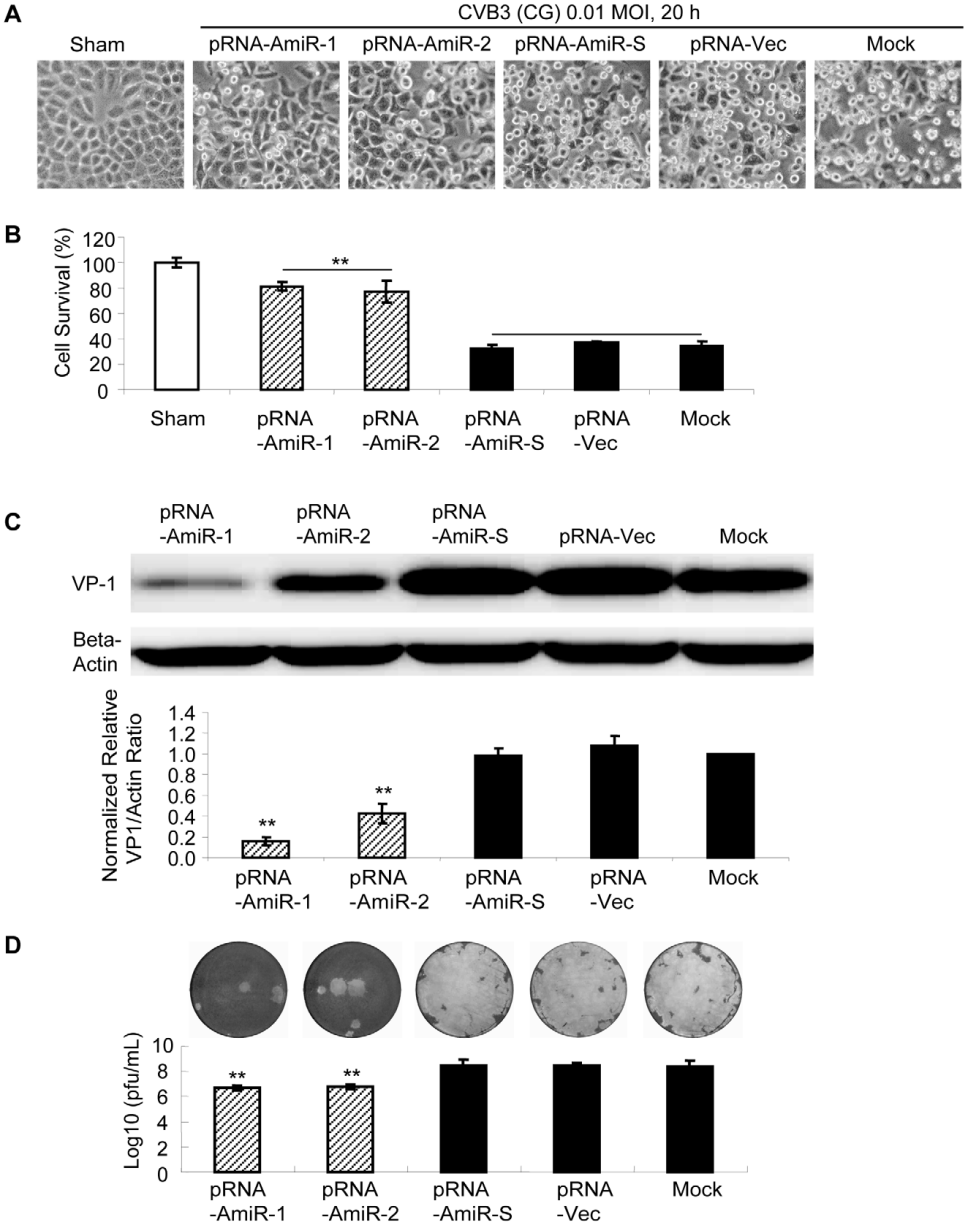
Anti-CVB3 (CG, low MOI) effect of pRNA-AmiR chimerics in HeLa cells. HeLa cells were transfected with different pRNA-AmiRs or mock-transfected with Oligofectamine™ only for 48 h and then infected with CVB3 (CG, 0.01 MOI 20 h) or sham-infected with PBS. Cell morphologies were analyzed by phase-contrast microscopy. Dying cells appeared rounding and detachment (A). Cell viability was measured by MTS assay (B), the viral VP-1 protein was detected by Western blot analysis (C) and viral particle formation was measured by plaque assay (D).

**Figure 8:**
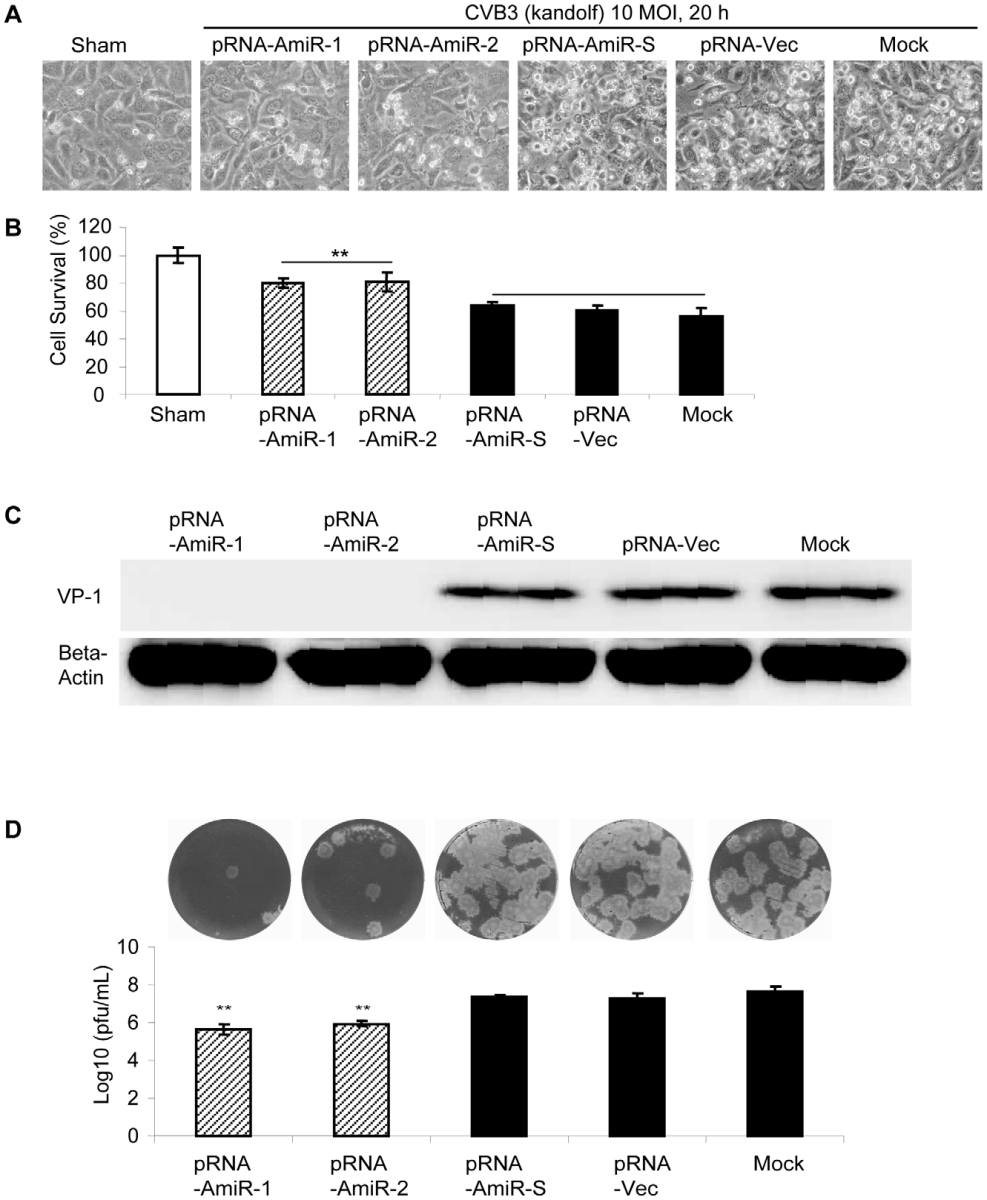
Anti-CVB3 (Kandolf) effect of pRNA-AmiR chimerics in HL-1 cells. Cardiomyocyte HL-1 cells were transfected with different pRNA-AmiRs or mock-transfected with Oligofectamine™ only for 48 h and then infected with CVB3 (Kandolf, 10 MOI 20 h) or sham-infected with PBS. Cell morphologies were analyzed by phase-contrast microscopy. Dying cells appeared rounding and detachment (A). Cell viability was measured by MTS assay (B), the viral VP-1 protein was detected by Western blot analysis (C) and viral particle formation was measured by plaque assay (D).

**Figure 9:**
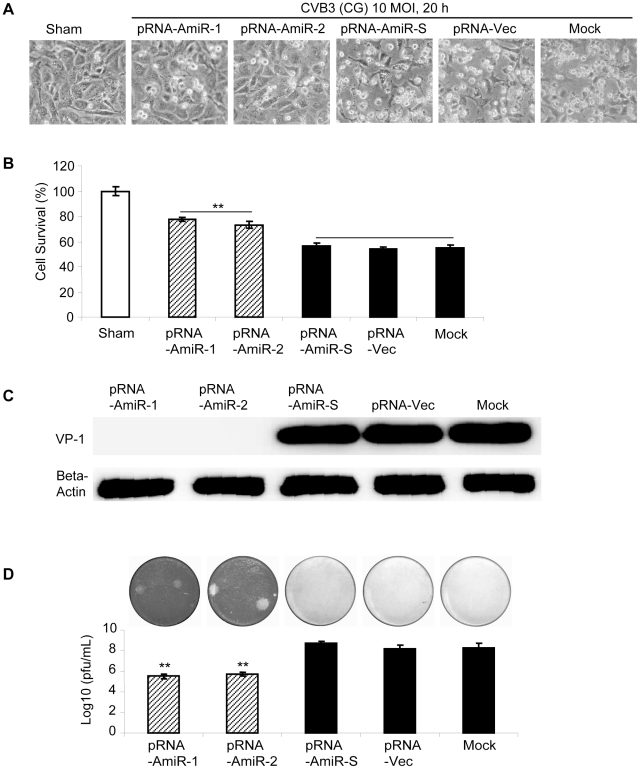
Anti-CVB3 (CG) effect of pRNA-AmiR chimerics in HL-1 cells. HL-1 cells were transfected with different pRNA-AmiRs or mock-transfected with Oligofectamine™ only for 48 h and then infected with CVB3 (CG, 10 MOI 20 h) or sham-infected with PBS. Cell morphologies were analyzed by phase-contrast microscopy. Dying cells appeared rounding and detachment (A). Cell viability was measured by MTS assay (B), the viral VP-1 protein was detected by Western blot analysis (C) and viral particle formation was measured by plaque assay (D).

To determine whether AmiRs still had anti-CVB3 activity after processing from the pRNA vector, we observed cell morphology and measured viral protein synthesis as well as viral particle release in the pRNA-AmiR transfected HeLa cells. Meanwhile, to confirm the anti-CVB3 effectiveness of the pRNA-AmiR in different CVB3 strains, we infected the cells using two viral strains (Kandolf and CG), respectively. We also used high and low viral titers (10 and 0.01 MOI) to determine the inhibitory effect of pRNA-AmiRs on cells infected for both long and short terms. Both cell morphologies and MTS cell viability assay showed that pRNA-AmiR-1 and pRNA-AmiR-2 effectively prevented the cells from death caused by CVB3 (Kandolf) infection. During the high titers of viral infection (10 MOI), as shown in Fig. 5A,and 5B, the living cells in plates treated with pRNA-AmiR-1 and pRNA-AmiR-2 at 8 h post infection were 80% and 70%, respectively; while only approximately 40% cells survived in other groups. The CVB3-induced caspase-3 cleavage, a widely used marker for cell apoptosis, was dramatically attenuated by the treatment of pRNA-AmiR-1 or pRNA-AmiR-2 (Fig. 5C). The antiviral effects of these AmiRs were further validated by Western blot analysis of VP-1 protein and plaque assay of the CVB3 particle formation. The data showed that substantial inhibition of VP-1 expression (Fig. 5D) and an approximately 1.5 log_10_ reduction of viral particle numbers (Fig. 5E) were observed in pRNA-AmiR-1 and pRNA-AmiR-2 transfected cells, respectively, as compared with the scrambled pRNA-AmiR-S, pRNA-Vec, and mock-tranfected controls. These results were more significant when infected the cells with a lower CVB3 titer (0.01 MOI). As shown in Fig. 6, an over 50% increase in cell survival, approximately 90% (pRNA-AmiR-1) or 60% (pRNA-AmiR-2) decrease in VP1 production and a 2-log_10_ reduction of viral progeny release were observed as compared to the control groups. The antiviral effect of the pRNA-AmiR was further confirmed by using another strain of CVB3 (CG) as shown in Fig. 7. To further determine the therapeutic potential of the pRNA-AmiR, we also perform the evaluation using HL-1 cells, a mouse cardiomyocyte cell line which is more closely related to the *in vivo* myocarditis model of CVB3 infection in heart. As shown in Fig. 8 and Fig. 9, the pRNA-AmiR-1 and pRNA-AmiR-2 in both viral strains were also able to decrease CVB3 induced cell death and inhibit CVB3 replication with about 1.5 log_10_ reduction of viral progeny release. In general, the antiviral effect of AmiRs was well preserved after linking to a pRNA, and the AmiRs were properly processed to functional miRNAs after delivery.

### Targeted delivery of pRNA-AmiRs chimerics via folate and its receptor interactions

**Figure 10:**
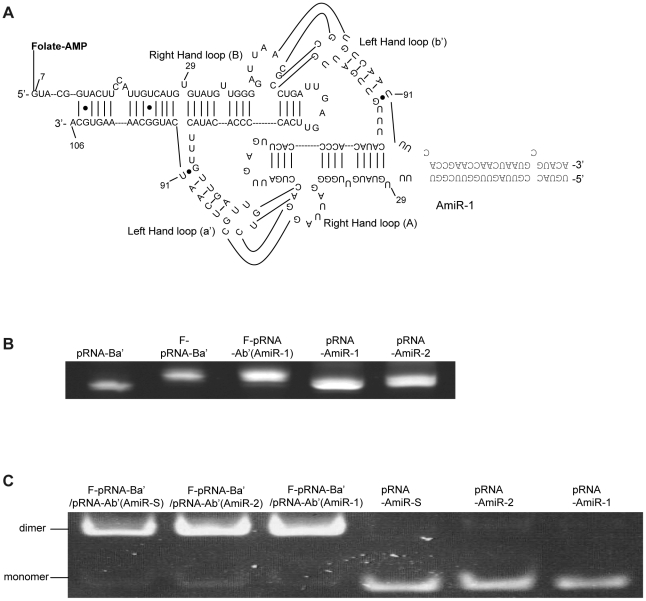
Preparation of folate-labeled pRNA-AmiR monomers and heterodimers. **A) Secondary structure of folate-labeled chimeric pRNA-AmiR heterodimer.** The 21-bp AmiR marked by a frame was covalently connected to a pRNA(A-b')(nt 29–91) vector to form pRNA-AmiR-1(A-b'). A pRNA(B-a') vector was labeled with folate and dimerized with pRNA-AmiR (A-b') by base-paring between the left- and right-hand loops of the two monomers. **B) Analysis of folate-labeled pRNA-AmiRs.** The *in vitro* transcription products of folate (F)-labeled and non-labeled pRNA-AmiRs were analyzed by denaturing urea-PAGE. **C) Analysis of folate-labeled pRNA heterodimers.** The folate labeled pRNA vector (F-pRNA-Ba') was dimerized with pRNA-AmiR. The dimerization products were analyzed by native PAGE. The dimers show a lower migration speed than the monomers.

pRNA has been employed to successful delivery siRNA to target cells [42], [44], [45], [65] by linking to a folate ligand. Effective binding and internalization of folate conjugated pRNA has been demonstrated by previous studies in our lab [42]. To determine whether folate-labeled pRNA (F-pRNA) was also applicable for AmiRs delivery, we conjugated folate to our pRNA-AmiR chimerics and incubated them with folate starved HeLa cells. In addition, since pRNA molecules are able to form dimers through the interaction of left- and right-hand loops [47], [48], [49], we also assembled the folate labeled pRNA-Vec and pRNA-AmiR chimerics together to form pRNA dimers. In order to construct the dimers, a pRNA (Ba') (nt 7–109) molecule with a left-hand loop a' (3′ C_85_C_84_U_83_G_82_) and a right-hand loop B (5′ A_45_C_46_G_47_C_48_) was conjugated to folate. Because all the pRNA-AmiR chimerics were designed based on the pRNA (Ab') vector which contained a right-hand loop A (5′ G_45_G_46_A_47_C_48_) and a left-hand loop b' (3′ U_84_G_83_C_82_G_81_), these chimerics can interact to the pRNA Ba' vector complementarily and form dimers (Fig. 10A). The folate-labeled pRNA-AmiR chimerics were analyzed by denaturing urea-PAGE and the heterodimers were confirmed by native PAGE in TBM buffer (Fig. 10B, and 10C). We constructed two monomers (F-pRNA-Ba' and F-pRNA-Ab'(AmiR-1)) and three heterodimers (F-pRNA-Ba'/pRNA-Ab'(AmiR-1), F-pRNA-Ba'/pRNA-Ab'(AmiR-2) and F-pRNA-Ba'/pRNA-Ab'(AmiR-S)) for this study.

**Figure 11:**
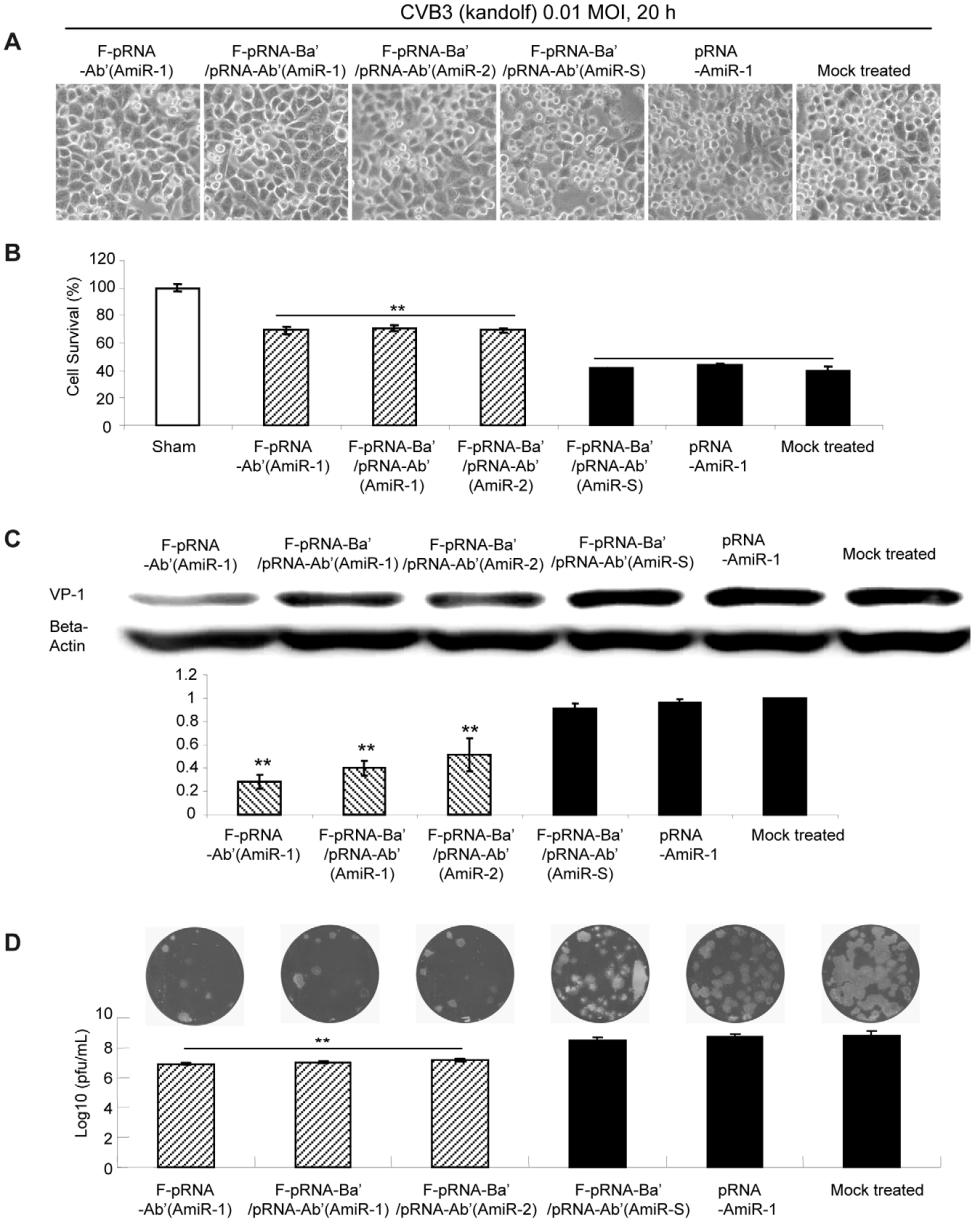
Antiviral effect of AmiRs delivered by folate-labeled pRNA-AmiR monomers and heterodimers. HeLa cells were treated with folate-labeled or non-labeled pRNA-AmiR monomers or heterodimers and infected with CVB3. The non-treated sample, F-pRNA-Ba'/pRNA-Ab'(AmiR-S) treated cells and non-labeled pRNA-AmiR-1 treated sample were used as three controls. Cell morphology was observed under a phase contrast microscope. Dying cells appeared rounding and detachment (A). Cell viability was also quantified by MTS assay (B). Anti-CVB3 effect was evaluated by Western blot analysis of VP-1 protein (C) and viral plaque assay of CVB3 particles (D).

To evaluate the antiviral effect of pRNA-AmiR chimerics delivered through folate-mediated internalization, we incubated (rather than transfected) the F-pRNA-Ab'(AmiR) monomers and heterodimers with HeLa cells for 8 h and then infected the cells with CVB3 for 20 h. Here we did not use HL-1cells as they can not be cultured in RPMI medium. We used three control groups to monitor the effectiveness and specificity of the chimerics: i) F-pRNA-Ba'/pRNA-Ab'(AmiR-S) control for the vector and non-specific AmiR sequences, ii) non-labeled pRNA-AmiR-1, control for the specificity of delivery via folate internalization and iii) the mock treated cells (binding solutions without any chimerics), a fundamental negative control. The cell morphologies and MTS assay indicated that more viable cells were present in culture treated with F-pRNA-Ab'(AmiR-1) (69%), F-pRNA-Ba'/pRNA-Ab'(AmiR-1) (71%) and F-pRNA-Ba'/pRNA-Ab'(AmiR-2) (69%) than all the control groups (around 40%) (Fig. 11A and 11B). In addition, Western blot showed that CVB3 VP1 synthesis was suppressed in F-pRNA-Ab'(AmiR-1) (0.3), F-pRNA-Ba'/pRNA-Ab'(AmiR-1) (0.4) and F-pRNA-Ba'/pRNA-Ab'(AmiR-2) (0.5) (Fig. 11C). Plauqe assay also showed about 1.3 log_10_ reduction of viral release in the three groups above (Fig. 11D). These results suggest that folate-mediated delivery of pRNA-AmiR is a powerful system for targeted delivery of therapeutic RNA molecules.

### pRNA-AmiRs targeting the 3′UTR of CVB3 tolerate mutations of the target

**Figure 12:**
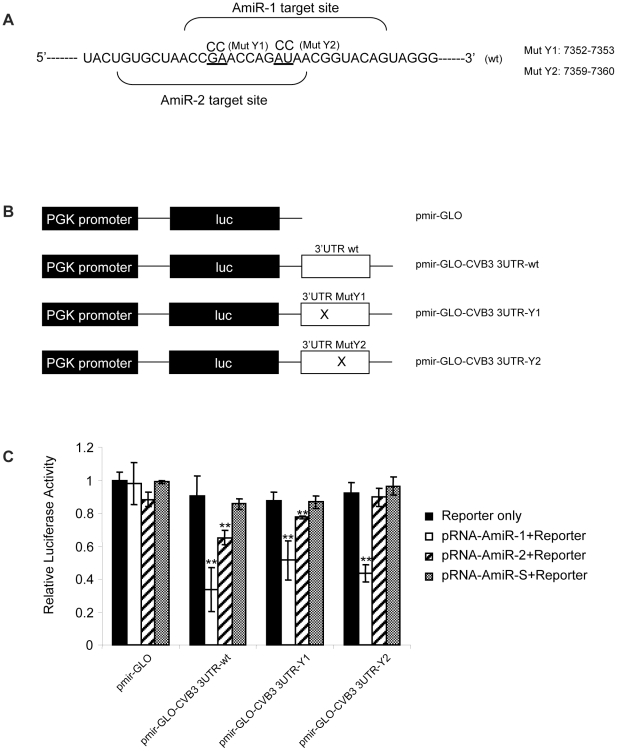
Luciferase reporter assay to determine the specific targeting and the tolerance of AmiRs to mutations. **A) Design of the CVB3 3′UTR mutants.** Part of the CVB3 3′UTR harboring the targeting sites of AmiR-1 and AmiR-2 was presented. Two mutants were designed by substitution of nucleotides which are underlined. The numbers indicate the positions of the nts within the 3′UTR of CVB3. B) **Schematic diagram of the pmir-GLO-CVB3-3′UTR reporter construct.** The CVB3 3′UTR sequences (wt or mut) were inserted after the 3′ end of firefly luciferase gene in the pmir-GLO vector. **C) Luciferase Assay of the targeting effect of pRNA-AmiR chimerics.** HeLa cells were co-transfected with pRNA-AmiR chimerics and luciferase reporter constructs containing wt or mutated CVB3 3′UTR. The cells transfected only with a given reporter construct listed in B) above served as a control in each specific group. Firefly and *Renilla* luciferase activities were detected using Dual-Glo luciferase analysis system 48 h after transfection. The ratio of luciferase activities (firefly/*Renilla*) was calculated and normalized to the one transfected only with empty pmir-GLO vector.

To test whether our pRNA-AmiRs could specifically target the 3′UTR of CVB3 RNA and their ability to tolerate mutations within the targets, we constructed luciferase reporters harboring either wt or mutated 3′UTR of the CVB3 genome. Because the secondary structure of the mRNA 3′UTR can affect the miRNA targeting [66], we inserted almost the whole CVB3 3′UTR sequence (nt 7306–nt 7399) instead of merely the target sites. To test whether the location of mutation would affect the outcome of targeting, two mutations were created. The first one (Mut-Y1) had two point mutations at nt 7352–7353, which complement to the 3′ region of AmiR-1 and the middle region of the AmiR-2, respectively. The second one (Mut-Y2) introduced two point mutations at nt 7359 to 7360, which are complementary to the middle region of AmiR-1 and the 5′ end region of AmiR-2, respectively (Fig. 12A and 12B). The luciferase reporter constructs were then co-transfected with pRNA-AmiR-1, pRNA-AmiR-2 or pRNA-AmiR-S. Luciferase activity measurements showed that both AmiR-1 and AmiR-2 could target the wt 3′UTR of CVB3 and reduced the reporter gene expression compared with pmir-GLO vector-only control, although AmiR-2 showed much lower inhibitory effect (30%) than AmiR-1 (65%) (Fig. 12C). When comparing the mutants, it was found that AmiR-1 still showed 45% and 50% inhibitory effect when targeting mutated sites at 5′end region (Mut-Y1) or middle region (Mut-Y2) of the AmiR-1 target sequence, respectively, though they were lower than the wt 3′UTR (65%, p<0.05). However, for AmiR-2, mutation at the 3′ end region (Mut-Y2) of the target (complementary to the seed sequence of miRNA) almost completely abolished the inhibitory effect; whereas, mutation at the middle region (Mut-Y1) of AmiR-2 target did not abolished the inhibitory effect of AmiR-2 (still achieved 20% inhibitory effect, but lower than wt 3′UTR (30%, p<0.05)). As expected, pRNA-AmiR-S did not show any targeting effect on the CVB3 3′UTR (Fig. 12C). These results suggest that AmiRs could regulate the gene expression in a similar manner as endogenous miRNAs and could preserve its inhibitory activity when a mutation occurs at the non-seed-match region of target sequences.

### The designed AmiRs and pRNA-AmiRs have little effect on IFN induction

**Figure 13:**
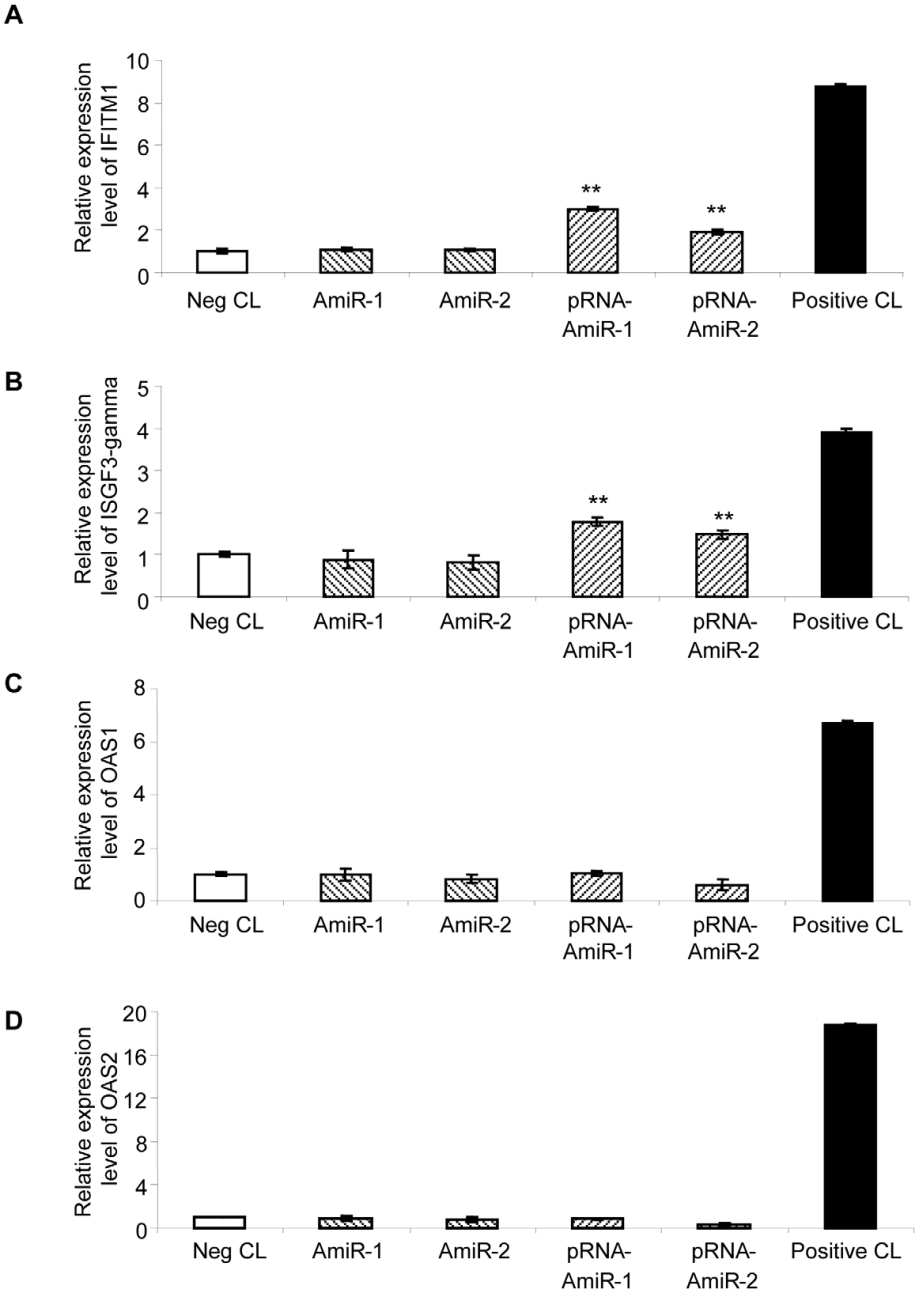
Detection of the IFN response in AmiR vector or pRNA-AmiR transfected HeLa cells. Total RNAs from HeLa cells transfected with pRNA vector or pRNA-AmiR were isolated and reverse transcribed to cDNA. Q-RT-PCR was conducted to detect the expression level of IFN response markers including IFITM1 (A), ISGF3-gamma (B), OAS1 (C) and OAS2 (D).

As one major concern of RNAi-based anti-viral therapy is the side effect caused by IFN induction after delivery of exogenous short RNAs [67], [68], we tested the IFN induction by the AmiR vectors and pRNA-AmiR transfection using IFN response detection kit. As showed in Fig. 13, AmiR-expressing vectors did not induce upregulation of any markers for IFN response and pRNA-AmiR transfection only lead to minimal increase in IFITM1 and ISGF3-gamma but showed no effect on induction of OAS1 and OAS2. These results indicate that the designed AmiR and pRNA-AmiR have very limited effect on IFN induction and may be good candidates for developing anti-CVB3 drugs.

## Discussion

One of the major limitations in RNAi-based viral inhibition is the viral escape due to target mutations. In this study, we designed artificial miRNAs targeting the 3′UTR of CVB3 genome, a crucial region for the viral replication, to inhibit CVB infection [37], [38]. By partially complementary targeting the 3′UTR, the AmiRs achieved promising anti-viral effect. This mode of action also resembles the natural miRNAs by imperfectly matching the targeting site of 3′UTR in the target genes. The capability of the designed AmiRs to tolerate mutations was further confirmed by introducing mismatches in the 3′UTR of CVB3, which highlighted the advantage of AmiR therapy in RNAi-based viral inhibition in terms of anti-mutation escape. To deliver the AmiR molecules into the cells in a specific manner, we conjugated the AmiRs to a pRNA vector labeled with folate, which, to the best of our knowledge, has not been tested for AmiR delivery. This F-pRNA-Ab'(AmiR) chimerics was delivered to the target cells via the interactions with the folate receptor, which is highly expressed in cancer cells. This is the first report to demonstrate that miRNA-like molecules can also be transported by pRNA conjugates and its biological activity is preserved during delivery. This novel targeted delivery system may be widely applicable to introduce other natural or artificial miRNAs into cancer cells for cancer study and treatment.

### AmiR target location and anti-CVB3 effect

Depending on the target regions in 3′UTR of CVB3 genome, we observed strong inhibitory effects on viral replication by AmiR-1 and AmiR-2, both of which target the big loop region (Y loop). These two AmiRs significantly improved the cell viability during CVB3 infection. No anti-CVB3 effect was found for AmiR-3 targeting the stem region of CVB3 3′UTR, which indicates that the secondary structures of the targeting sites play an important role in affecting the targeting of AmiRs. The loop region with more open spatial configurations may be easier for the AmiRs to hybridize; while the stem region may not provide a convenient access for AmiRs. Consistent with our results, previous studies also suggested that the interactions between miRNAs and their targets would be affected by the target structure, and the open loop regions may be more accessible to the miRNA targeting [66], [69]. This would provide important clues for the future designing of anti-viral AmiRs.

### Tolerance of siRNA and AmiR to viral genomic mutation

**Figure 14:**
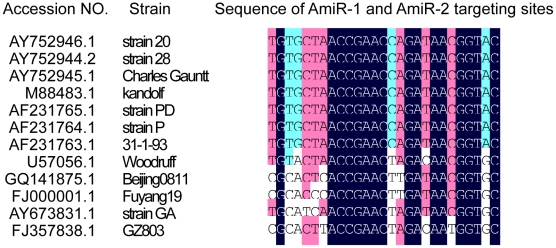
Comparison of AmiR-1 and AmiR-2 targeting sites in different CVB3 strains. CVB3 whole genome sequences were obtained from Gene Bank and the regions targeted by AmiR-1 and AmiR-2 were compared using DNAMAN.

It has been reported that siRNAs may tolerate the mutations in a certain extent but they have strong mismatch discriminations [70]. The middle region of the siRNA is very sensitive to target mutations while the 5′ and 3′ ends are relatively tolerable. Previous studies in our laboratory using siRNA targeting CVB3 2A protease gene also found that the central region of siRNAs is very sensitive to target mutation [10]. In our present study, the designed AmiRs showed robust anti-viral effect with partial complementation to the 3′UTR of CVB3 genome. They are tolerable to the mismatches in their middle and 3′ region but not the 5′ region though the antiviral effect was slightly reduced due to the mismatches (Fig. 12C). Previous studies on the targeting principles of cellular miRNA have revealed that 5′ seeding region of miRNA is crucial for miRNA targeting and the complementation at the 3′ region may be beneficial, but not always necessary, for the specificity of miRNAs [30]. On the other hand, the complementarity in the middle regions of miRNAs with its target is related to the regulatory mechanism of miRNAs [30], [60]. Mismatches between miRNAs and their targets in the middle region may prevent the cleavage of the targets. However, most endogenous and engineered miRNAs are still effective with mismatches to their targets in the central region, indicating this region is not as essential as the 5′ or 3′ end in terms of targeting effect [30], [60]. Other studies [5], [13], [14] and our data suggest that AmiRs that mimic endogenous miRNAs probably use the same mechanism of action. The mismatches in the central regions only slightly reduced the targeting effect while the ones in the 5′ seeding region almost abolished the inhibitory function. Our results provide further support that siRNA and miRNA are distinct from each other in terms of mutation tolerance. This suggests that the combination of siRNA and miRNA may prevent the viral escape caused by mutations. In addition, the designed AmiR-1 and AmiR-2 showed inhibitory effect on both Kandolf and CG strains of CVB3. We further found that the AmiR-1 and AmiR-2 targeting sites are highly conserved in the genomes of various CVB3 strains after bioinformatic analysis using DNAMAN (Lynnon BioSoft). Seven of the 12 strains are exact the same and the other 5 share fairly high homology in their 3′UTRs (Fig. 14). Thus, the designed AmiRs likely have a broad spectrum of anti-CVB3 activity for various CVB3 strains.

### Targeting of AS-ODN, siRNA and AmiR

Our previous studies designed AS-ODNs [39] and siRNAs [10] targeting different regions of CVB3 genome to inhibit the viral replication. By comparing these data with the present AmiR study, we found that different anti-viral agents may tend to target different regions of the viral genome. Our AS-ODNs were designed to target either the 5′ or 3′UTR region of CVB3 genome and it seems that most of the effective ones were targeting the 5′UTR and only one AS-ODN targeting the end of the 3′UTR was effective [39]; while for the siRNAs, almost all of the effective ones were targeting the coding region but not the 5′ or 3′UTR [10]. In case of AmiRs, we designed three AmiRs targeting the 3′UTR and two of them demonstrated anti-CVB3 effect. Other studies using AmiRs targeting HIV-1 genomic RNA showed that AmiRs targeting the 3′UTR had stronger antiviral activity than the one targeting the coding regions [5], [13].

Another interesting point here is that even though targeting the same site, AS-ODNs, siRNAs and AmiRs may achieve different antiviral effects. One of the AS-ODNs designed to target nt 7342–7361 in CVB3 3′UTR showed no antiviral effect [39], while in the present study, AmiR-2 targeting nearly the same site (nt 7342–7362) exhibited inhibition on CVB3 replication. For targeting within the 5′UTR of CVB3, one of the AS-ODNs targeting nt 733–752 efficiently suppressed the CVB3 translation and replication [39]; while the designed siRNA targeting the same region (nt 733–751) showed little effect on CVB3 infection [10]. Although we can not exclude the possibility that these differences were caused by one nucleotide shift in the targeting sites, it seems more likely that these three different types of pro-drugs may function through distinct modes and thus showed different targeting effect in the same targeting sites.

### Specific delivery of AmiRs using pRNA to treat CVB3 infection

The pRNA vector is a novel system for delivery of therapeutic molecules like siRNAs [42], [45], [46], [47], [48]. However, this strategy has not been investigated on AmiRs. The replacement of the helical region of pRNA with two complementary strands of designed nucleotide sequences, like siRNA, does not influence its folding and structure [55], [71]. Although miRNA duplexes are usually not totally base-paired as siRNA, the helical region of the original pRNA molecule also contains mismatches. Based on these characteristics, it is also possible to replace the helical region of the pRNA with miRNA duplex sequences for delivering miRNAs. This study showed that the anti-CVB3 function of AmiRs was preserved after covalently linking to a pRNA vector. The folate-labeled pRNA-AmiR chimeric monomers, as well as heterodimers, were then successfully delivered into HeLa cells via the folate receptor and processed into functional, mature AmiRs. The AmiRs delivered by this strategy also showed anti-CVB3 effect. Thus our study is the first successful attempt to deliver miRNAs to target cells using the pRNA vector.

The application of pRNA technology in treatment of CVB3 infection and viral myocarditis has several advantages. First, with its nanoscale size, pRNA has lower immunogenicity than other viral vectors [48], [72]. Second, pRNA monomers possess the unique capabilities to auto-assemble to dimmers, trimers and even hexamers [47], [48], which stabilizes the carried drugs and provides the opportunities for simultaneous delivery of several therapeutic agents as well as the specific ligands for targeted delivery [73]. In addition, the drug delivery system using pRNA is protein free, resulting in lower antibody-inducing activity that is essential for repeated administration to treat chronic diseases like viral myocarditis [74]. The previous attempts to deliver chemically modified siRNA only last 15–45 min in the body and often trigger undesired immune response [75], [76]. After linking to pRNA vector, the siRNA particles can achieve a half-life of 5–10 h with little immune response or other toxicity observed in both *in vitro* and *in vivo* administration [77].

However, there are still several challenges. The first one is to increase the efficiency of delivery of pRNA-AmiR via ligand and receptor interactions. This is based on the observation that pRNA-AmiRs delivered by folate mediated internalization showed lower anti-CVB3 effect than the one transfected by Oligofectamine™. The second one is to search for other ligands such as RNA aptamers, peptide or chemical ligands specific for the heart delivery [78]. In this initial study, we used HeLa cells, a folate-receptor positive cancer cell line and also a CVB3 susceptible host, for treating CVB3 infection. For our final goal, we need to discover new ligands to specifically deliver the drug to the heart to treat CVB3 infection and viral myocarditis.

In conclusion, our designed AmiRs targeting the 3′UTR of CVB3 can inhibit viral replication and tolerate mutations in the middle regions of target sites. This suggests the potential of AmiRs to reduce drug resistance caused by viral escape mutations and the promise to deliver AmiRs using folate-conjugated pRNA vectors linked with a specific ligand. This strategy may be further developed as a system for RNAi-based drug design and delivery.

## References

[1] Drory Y, Turetz Y, Hiss Y, Lev B, Fisman EZ (1991). Sudden unexpected death in persons less than 40 years of age.. Am J Cardiol.

[2] Huber SA, Gauntt CJ, Sakkinen P (1998). Enteroviruses and myocarditis: viral pathogenesis through replication, cytokine induction, and immunopathogenicity.. Adv Virus Res.

[3] Tan FL, Yin JQ (2004). RNAi, a new therapeutic strategy against viral infection.. Cell Res.

[4] Chen Y, Cheng G, Mahato RI (2008). RNAi for treating hepatitis B viral infection.. Pharm Res.

[5] Liu YP, Gruber J, Haasnoot J, Konstantinova P, Berkhout B (2009). RNAi-mediated inhibition of HIV-1 by targeting partially complementary viral sequences.. Nucleic Acids Res.

[6] Gitlin L, Stone JK, Andino R (2005). Poliovirus escape from RNA interference: short interfering RNA-target recognition and implications for therapeutic approaches.. J Virol.

[7] Ge Q, Eisen HN, Chen J (2004). Use of siRNAs to prevent and treat influenza virus infection.. Virus Res.

[8] Kapadia SB, Brideau-Andersen A, Chisari FV (2003). Interference of hepatitis C virus RNA replication by short interfering RNAs.. Proc Natl Acad Sci U S A.

[9] Novina CD, Murray MF, Dykxhoorn DM, Beresford PJ, Riess J (2002). siRNA-directed inhibition of HIV-1 infection.. Nat Med.

[10] Yuan J, Cheung PK, Zhang HM, Chau D, Yang D (2005). Inhibition of coxsackievirus B3 replication by small interfering RNAs requires perfect sequence match in the central region of the viral positive strand.. J Virol.

[11] Ahn J, Jun ES, Lee HS, Yoon SY, Kim D (2005). A small interfering RNA targeting coxsackievirus B3 protects permissive HeLa cells from viral challenge.. J Virol.

[12] Chen JB, Xu CF, Yu Y, Li L, Li SJ (2006). Inhibitory effect of CVB3-VP1 siRNA on CVB3 replication.. Xi Bao Yu Fen Zi Mian Yi Xue Za Zhi.

[13] Son J, Uchil PD, Kim YB, Shankar P, Kumar P (2008). Effective suppression of HIV-1 by artificial bispecific miRNA targeting conserved sequences with tolerance for wobble base-pairing.. Biochem Biophys Res Commun.

[14] Israsena N, Supavonwong P, Ratanasetyuth N, Khawplod P, Hemachudha T (2009). Inhibition of rabies virus replication by multiple artificial microRNAs.. Antiviral Res.

[15] Tang G (2005). siRNA and miRNA: an insight into RISCs.. Trends Biochem Sci.

[16] Drake JW, Holland JJ (1999). Mutation rates among RNA viruses.. Proc Natl Acad Sci U S A.

[17] Sanjuan R, Nebot MR, Chirico N, Mansky LM, Belshaw R Viral mutation rates.. J Virol.

[18] Darlix JL, Spahr PF (1983). High spontaneous mutation rate of Rous sarcoma virus demonstrated by direct sequencing of the RNA genome.. Nucleic Acids Res.

[19] Crotty S, Andino R (2002). Implications of high RNA virus mutation rates: lethal mutagenesis and the antiviral drug ribavirin.. Microbes Infect.

[20] Gitlin L, Karelsky S, Andino R (2002). Short interfering RNA confers intracellular antiviral immunity in human cells.. Nature.

[21] Machida K, Cheng KT, Sung VM, Shimodaira S, Lindsay KL (2004). Hepatitis C virus induces a mutator phenotype: enhanced mutations of immunoglobulin and protooncogenes.. Proc Natl Acad Sci U S A.

[22] Brackney DE, Beane JE, Ebel GD (2009). RNAi targeting of West Nile virus in mosquito midguts promotes virus diversification.. PLoS Pathog.

[23] Merl S, Wessely R (2007). Anti-coxsackieviral efficacy of RNA interference is highly dependent on genomic target selection and emergence of escape mutants.. Oligonucleotides.

[24] Lee HS, Ahn J, Jee Y, Seo IS, Jeon EJ (2007). Universal and mutation-resistant anti-enteroviral activity: potency of small interfering RNA complementary to the conserved cis-acting replication element within the enterovirus coding region.. J Gen Virol.

[25] Schubert S, Grunert HP, Zeichhardt H, Werk D, Erdmann VA (2005). Maintaining inhibition: siRNA double expression vectors against coxsackieviral RNAs.. J Mol Biol.

[26] Han J, Lee Y, Yeom KH, Kim YK, Jin H (2004). The Drosha-DGCR8 complex in primary microRNA processing.. Genes Dev.

[27] Lee YS, Nakahara K, Pham JW, Kim K, He Z (2004). Distinct roles for Drosophila Dicer-1 and Dicer-2 in the siRNA/miRNA silencing pathways.. Cell.

[28] Macrae IJ, Zhou K, Li F, Repic A, Brooks AN (2006). Structural basis for double-stranded RNA processing by Dicer.. Science.

[29] Engels BM, Hutvagner G (2006). Principles and effects of microRNA-mediated post-transcriptional gene regulation.. Oncogene.

[30] Brennecke J, Stark A, Russell RB, Cohen SM (2005). Principles of microRNA-target recognition.. PLoS Biol.

[31] Fechner H, Sipo I, Westermann D, Pinkert S, Wang X (2008). Cardiac-targeted RNA interference mediated by an AAV9 vector improves cardiac function in coxsackievirus B3 cardiomyopathy.. J Mol Med.

[32] Merl S, Michaelis C, Jaschke B, Vorpahl M, Seidl S (2005). Targeting 2A protease by RNA interference attenuates coxsackieviral cytopathogenicity and promotes survival in highly susceptible mice.. Circulation.

[33] Kim YJ, Ahn J, Jeung SY, Kim DS, Na HN (2008). Recombinant lentivirus-delivered short hairpin RNAs targeted to conserved coxsackievirus sequences protect against viral myocarditis and improve survival rate in an animal model.. Virus Genes.

[34] McBride JL, Boudreau RL, Harper SQ, Staber PD, Monteys AM (2008). Artificial miRNAs mitigate shRNA-mediated toxicity in the brain: implications for the therapeutic development of RNAi.. Proc Natl Acad Sci U S A.

[35] Boudreau RL, Martins I, Davidson BL (2009). Artificial microRNAs as siRNA shuttles: improved safety as compared to shRNAs in vitro and in vivo.. Mol Ther.

[36] Dunn JJ, Chapman NM, Tracy S, Romero JR (2000). Genomic determinants of cardiovirulence in coxsackievirus B3 clinical isolates: localization to the 5′ nontranslated region.. J Virol.

[37] Melchers WJ, Hoenderop JG, Bruins Slot HJ, Pleij CW, Pilipenko EV (1997). Kissing of the two predominant hairpin loops in the coxsackie B virus 3′ untranslated region is the essential structural feature of the origin of replication required for negative-strand RNA synthesis.. J Virol.

[38] Cheung P, Lim T, Yuan J, Zhang M, Chau D (2007). Specific interaction of HeLa cell proteins with coxsackievirus B3 3′UTR: La autoantigen binds the 3′ and 5′UTR independently of the poly(A) tail.. Cell Microbiol.

[39] Wang A, Cheung PK, Zhang H, Carthy CM, Bohunek L (2001). Specific inhibition of coxsackievirus B3 translation and replication by phosphorothioate antisense oligodeoxynucleotides.. Antimicrob Agents Chemother.

[40] De Guire V, Caron M, Scott N, Menard C, Gaumont-Leclerc MF (2010). Designing small multiple-target artificial RNAs.. Nucleic Acids Res.

[41] Tsuda N, Ishiyama S, Li Y, Ioannides CG, Abbruzzese JL (2006). Synthetic microRNA designed to target glioma-associated antigen 1 transcription factor inhibits division and induces late apoptosis in pancreatic tumor cells.. Clin Cancer Res.

[42] Zhang HM, Su Y, Guo S, Yuan J, Lim T (2009). Targeted delivery of anti-coxsackievirus siRNAs using ligand-conjugated packaging RNAs.. Antiviral Res.

[43] Khaled A, Guo S, Li F, Guo P (2005). Controllable self-assembly of nanoparticles for specific delivery of multiple therapeutic molecules to cancer cells using RNA nanotechnology.. Nano Lett.

[44] Li L, Liu J, Diao Z, Shu D, Guo P (2009). Evaluation of specific delivery of chimeric phi29 pRNA/siRNA nanoparticles to multiple tumor cells.. Mol Biosyst.

[45] Guo S, Huang F, Guo P (2006). Construction of folate-conjugated pRNA of bacteriophage phi29 DNA packaging motor for delivery of chimeric siRNA to nasopharyngeal carcinoma cells.. Gene Ther.

[46] Guo PX, Erickson S, Anderson D (1987). A small viral RNA is required for in vitro packaging of bacteriophage phi 29 DNA.. Science.

[47] Guo P, Zhang C, Chen C, Garver K, Trottier M (1998). Inter-RNA interaction of phage phi29 pRNA to form a hexameric complex for viral DNA transportation.. Mol Cell.

[48] Guo P (2002). Structure and function of phi29 hexameric RNA that drives the viral DNA packaging motor: review.. Prog Nucleic Acid Res Mol Biol.

[49] Shu D, Huang LP, Hoeprich S, Guo P (2003). Construction of phi29 DNA-packaging RNA monomers, dimers, and trimers with variable sizes and shapes as potential parts for nanodevices.. J Nanosci Nanotechnol.

[50] Guo P The emerging field of RNA nanotechnology.. Nat Nanotechnol.

[51] Weitman SD, Lark RH, Coney LR, Fort DW, Frasca V (1992). Distribution of the folate receptor GP38 in normal and malignant cell lines and tissues.. Cancer Res.

[52] Xia W, Hilgenbrink AR, Matteson EL, Lockwood MB, Cheng JX (2009). A functional folate receptor is induced during macrophage activation and can be used to target drugs to activated macrophages.. Blood.

[53] Tran T, Shatnawi A, Zheng X, Kelley KM, Ratnam M (2005). Enhancement of folate receptor alpha expression in tumor cells through the glucocorticoid receptor: a promising means to improved tumor detection and targeting.. Cancer Res.

[54] Claycomb WC, Lanson NA, Stallworth BS, Egeland DB, Delcarpio JB (1998). HL-1 cells: a cardiac muscle cell line that contracts and retains phenotypic characteristics of the adult cardiomyocyte.. Proc Natl Acad Sci U S A.

[55] Zhang C, Tellinghuisen T, Guo P (1995). Confirmation of the helical structure of the 5′/3′ termini of the essential DNA packaging pRNA of phage phi 29.. RNA.

[56] Chen C, Sheng S, Shao Z, Guo P (2000). A dimer as a building block in assembling RNA. A hexamer that gears bacterial virus phi29 DNA-translocating machinery.. J Biol Chem.

[57] Chen C, Ridzon DA, Broomer AJ, Zhou Z, Lee DH (2005). Real-time quantification of microRNAs by stem-loop RT-PCR.. Nucleic Acids Res.

[58] Yuan J, Cheung PK, Zhang H, Chau D, Yanagawa B (2004). A phosphorothioate antisense oligodeoxynucleotide specifically inhibits coxsackievirus B3 replication in cardiomyocytes and mouse hearts.. Lab Invest.

[59] Lai EC (2002). Micro RNAs are complementary to 3′ UTR sequence motifs that mediate negative post-transcriptional regulation.. Nat Genet.

[60] Brodersen P, Voinnet O (2009). Revisiting the principles of microRNA target recognition and mode of action.. Nat Rev Mol Cell Biol.

[61] Ye W, Lv Q, Wong CK, Hu S, Fu C (2008). The effect of central loops in miRNA:MRE duplexes on the efficiency of miRNA-mediated gene regulation.. PLoS One.

[62] Zeng Y, Wagner EJ, Cullen BR (2002). Both natural and designed micro RNAs can inhibit the expression of cognate mRNAs when expressed in human cells.. Mol Cell.

[63] Parizotto EA, Dunoyer P, Rahm N, Himber C, Voinnet O (2004). In vivo investigation of the transcription, processing, endonucleolytic activity, and functional relevance of the spatial distribution of a plant miRNA.. Genes Dev.

[64] Kelley M, Birmingham A, Karpilow J, Khvorova A, Sullivan K (2010). Micro-RNA Scaffolds, Non-naturally Occurring Micro-RNAs, and Methods for Optimizing Non-naturally Occurring Micro-RNAs.

[65] Tarapore P, Shu Y, Guo P, Ho SM (2010). Application of Phi29 Motor pRNA for Targeted Therapeutic Delivery of siRNA Silencing Metallothionein-IIA and Survivin in Ovarian Cancers.. Mol Ther.

[66] Long D, Chan CY, Ding Y (2008). Analysis of microRNA-target interactions by a target structure based hybridization model.. Pac Symp Biocomput.

[67] Ma Z, Li J, He F, Wilson A, Pitt B (2005). Cationic lipids enhance siRNA-mediated interferon response in mice.. Biochem Biophys Res Commun.

[68] Reynolds A, Anderson EM, Vermeulen A, Fedorov Y, Robinson K (2006). Induction of the interferon response by siRNA is cell type- and duplex length-dependent.. RNA.

[69] Kertesz M, Iovino N, Unnerstall U, Gaul U, Segal E (2007). The role of site accessibility in microRNA target recognition.. Nat Genet.

[70] Huang H, Qiao R, Zhao D, Zhang T, Li Y (2009). Profiling of mismatch discrimination in RNAi enabled rational design of allele-specific siRNAs.. Nucleic Acids Res.

[71] Chen C, Zhang C, Guo P (1999). Sequence requirement for hand-in-hand interaction in formation of RNA dimers and hexamers to gear phi29 DNA translocation motor.. RNA.

[72] Guo P (2005). Bacterial virus phi29 DNA-packaging motor and its potential applications in gene therapy and nanotechnology.. Methods Mol Biol.

[73] Ye X, Yang D (2009). Recent advances in biological strategies for targeted drug delivery.. Cardiovasc Hematol Disord Drug Targets.

[74] Shu Y, Cinier M, Shu D, Guo P Assembly of multifunctional phi29 pRNA nanoparticles for specific delivery of siRNA and other therapeutics to targeted cells.. Methods.

[75] Soutschek J, Akinc A, Bramlage B, Charisse K, Constien R (2004). Therapeutic silencing of an endogenous gene by systemic administration of modified siRNAs.. Nature.

[76] Marques JT, Williams BR (2005). Activation of the mammalian immune system by siRNAs.. Nat Biotechnol.

[77] Abdelmawla S, Guo S, Zhang L, Pulukuri SM, Patankar P Pharmacological Characterization of Chemically Synthesized Monomeric phi29 pRNA Nanoparticles for Systemic Delivery.. Mol Ther.

[78] Parker N, Turk MJ, Westrick E, Lewis JD, Low PS (2005). Folate receptor expression in carcinomas and normal tissues determined by a quantitative radioligand binding assay.. Anal Biochem.

